# Complete uranium bioreduction in 48 hours: Synergistic electron transfer in a synthetic microbial consortium

**DOI:** 10.1016/j.ese.2025.100629

**Published:** 2025-10-21

**Authors:** Xizi Long, Yuanyuan Jiang, Zhaozhong Zhu, Yu Li, Nan Hu, Junzhan Hong, Hui Wang, Fei Yang

**Affiliations:** a The Key Laboratory of Typical Environmental Pollution and Health Hazards of Hunan Province, School of Public Health, University of South China, Hengyang, 421001, China; b Department of Municipal and Environmental Engineering, Faculty of Water Resources and Hydroelectric Engineering, Xi'an University of Technology, Xi'an, 710048, China; c Key Discipline Laboratory in Uranium Mining and Hydrometallurgy, University of South China, Hengyang, Hunan, 421001, China; d Hunan Provincial Key Laboratory of Geochemical Processes and Resource Environmental Effects, Geophysical and Geochemical Survey Institute of Hunan, Changsha, Hunan, 410014, China

**Keywords:** Uranium bioremediation, Synthetic microbial community, Extracellular electron transfer, eDNA, Pyocyanin

## Abstract

Uranium contamination from mining and natural sources poses a major environmental and health risk, as soluble uranium U(VI) readily migrates through groundwater systems. Microbial reduction to insoluble U(IV) via dissimilatory metal-reducing bacteria offers a sustainable remediation method, relying on extracellular electron transfer (EET) to shuttle electrons to extracellular acceptors. *Shewanella oneidensis* MR-1 (*S*.MR-1) serves as a model organism for this process, but its EET efficiency is hindered by limited endogenous redox mediators and biofilm conductivity. Despite advances in genetic engineering, the potential of synthetic microbial communities to enhance EET through interspecies interactions remains underexplored. Here we show a synthetic consortium comprising *S*.MR-1 and a non-U-reducing isolate, *Pseudomonas aeruginosa* LXZ1 (*P*.LXZ1), that fully reduces U(VI) within 48 h, compared to only 60 % reduction by *S*.MR-1 alone. This enhancement stems from *P*.LXZ1-secreted pyocyanin, which binds selectively to *S*.MR-1's outer-membrane cytochrome OmcA, shifting its redox potential to facilitate directional electron flow along a thermodynamic gradient. Concurrently, conductive extracellular DNA released by *P*. LXZ1 promotes electron transport and aggregate formation, as evidenced by electrochemical assays, transcriptomics, and molecular dynamics simulations. These synergistic mechanisms alleviate proton-transfer limitations and upregulate metabolic pathways, boosting overall EET rates. By harnessing natural microbial cooperation, this approach provides insights into community-driven metal reduction and paves the way for efficient, scalable bioremediation strategies in contaminated sites.

## Highlights

• A synthetic microbial community (*syncoms*) improves uranium bio-reducing efficiency.
• eDNA from *P. aeruginosa* LXZ1 promotes *syncoms* biofilm formation and boosts electron transfer.
• Pyocyanin excreted by *P. aeruginosa* LXZ1 enhances electron transfer via OmcA in *S. oneidensis* MR-1.
• *Syncoms* promote gene expression related to electron transfer and energy metabolism in *S. oneidensis* MR-1.

## Graphical abstract

**Figure:**
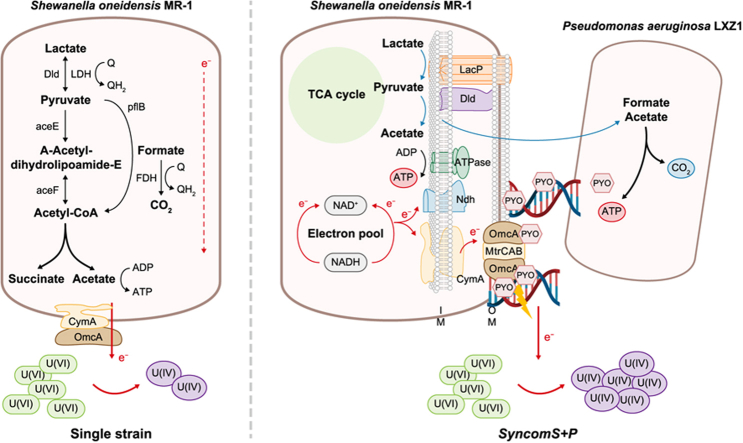

## 1 Introduction

The global spread of uranium (U) contamination, stemming from both natural and anthropogenic sources, has emerged as a critical environmental concern. U-bearing minerals, subjected to weathering and erosion, can release uranium into aquatic systems, often raising groundwater U(VI) concentrations beyond regulatory limits [1]. In parallel, intensified human activities, particularly uranium mining, have elevated soil uranium levels to 1000 mg kg^−1^ in affected areas [2], increasing ecological and health risks. Given the high solubility and mobility of hexavalent U(VI) [3], strategies aimed at reducing it to insoluble tetravalent U(IV) have become central to sustainable remediation efforts. Among these, the biological reduction of U(VI) using dissimilatory metal-reducing bacteria (DMRB) offers a promising pathway. *Shewanella oneidensis* MR-1 (*S*.MR-1), a model DMRB, reduces extracellular U(VI) through extracellular electron transfer (EET) mediated by *C*-type cytochromes and redox mediators such as riboflavin (RF) and flavin mononucleotide (FMN) [4,5]. Critical research has indicated that the U(VI) reduction efficacy of *Shewanella* sp. relies on mediators, with approximately 70 % of the EET attributed to self-secreted RF and FMN [6]. However, the *in situ* production of flavins within *S. oneidensis* MR-1 biofilms is limited to a maximum of only ∼250 nM, which significantly constrains the overall EET capacity and thereby hampers efficient U(VI) reduction [7].

Despite laborious flavin-based gene editing strategies to enhance EET and improve uranium removal efficiency [8], recent advances in the engineering of synthetic microbial communities (*SynComs*) from indigenous soil microbiota offer a promising alternative to overcome the intrinsic limitations of single-strain systems. These *SynComs* can enhance metabolic versatility, stabilize biofilm formation, and ultimately simplify the complex steps often required in synthetic biology-based bioremediation approaches [9]. However, the precise contributions of *SynComs* to uranium removal remain poorly understood.

Phenazines, a well-characterized class of redox–active metabolites, are widely synthesized by diverse environmental microbiomes [10,11], and are key contributors to EET in current-generating microbial consortia [12]. *Pseudomonas* species are the most prominent producers, secreting a suite of phenazine derivatives at 100 μM levels. Moreover, extracellular DNA (eDNA) produced by *Pseudomonas* sp.*,* traditionally viewed as a structural component within biofilms, has recently gained attention as a functional matrix that facilitates redox processes and reinforces the conductive properties of microbial biofilms [13]. However, its specific role in promoting uranium reduction through enhanced EET has yet to be systematically explored. Collectively, these findings suggest that achieving efficient U(VI) bio-reduction necessitates multidimensional improvements—namely, the increased production of redox mediators, the augmentation of metabolic activity, and the promotion of biofilm conductivity [14].

Here, we report a cooperative synthetic microbial system composed of *S*.MR-1 and a newly isolated *Pseudomonas aeruginosa* strain LXZ1 (*P*.LXZ1), derived from a uranium tailings reservoir. We demonstrate that *P*. LXZ1 actively secretes phenazine and releases eDNA into the biofilm matrix, jointly enhancing EET and promoting the reduction of soluble U(VI). Through a combination of electrochemical analyses, transcriptomic profiling, and molecular dynamics simulations, we systematically dissect the mechanistic roles of phenazines and eDNA in mediating redox dynamics and biofilm conductivity. The integration of *P*. LXZ1 into the consortium significantly accelerates U(VI) reduction by forming a flavoprotein complex and stabilizing conductive biofilm networks. Overall, our findings reveal a previously underappreciated synergy between phenazine biosynthesis and eDNA-mediated matrix formation within synthetic microbial consortia, highlighting a powerful strategy for developing robust, scalable, and sustainable bioremediation systems for targeting uranium contamination.

## 2 Materials and methods

### 2.1 Microbial sampling and cultivation conditions

*S*.MR-1 and mutant strains (Δ*omcA,* Δ*mtrC,* and Δ*cymA*) were cultivated overnight with LB medium (Sangon Biotech, China) at 180 rpm and 30 °C in 50 mL centrifuge tubes. Cells were subsequently harvested and washed twice with defined medium (DM) supplemented with 10 mM sodium lactate (DM-L) [15]. The composition of DM was as follows: NaHCO_3_, 2.5 g L^−1^; CaCl_2_·2H_2_O, 0.08 g L^−1^; NH_4_Cl, 1.0 g L^−1^; NaCl, 10 g L^−1^; MgCl_2_·6H_2_O, 0.2 g L^−1^; yeast extract, 0.5 g L^−1^; and 4-(2-hydroxyethyl)-1-piperazineethanesulfonic acid (HEPES, Sangon Biotech, China), 7.2 g L^−1^. The method used to construct mutants has been described in previous studies [16]. The *P*. LXZ1 strain was isolated from soil samples collected at the No.712 uranium mining area in Hengyang, Hunan Province, China, where the average uranium concentration in the topsoil exceeds the national background level by more than fourfold [17]. Taxonomy analysis of the collected soil microbial community revealed a relatively high abundance of *P*. *aeruginosa* [17].

### 2.2 Uranium reduction by *SynComs*

A stock solution of 800 mg L^−1^ uranyl acetate (ACMEC Tech, China) was diluted with DM-L to yield an experimental U(VI) solution at a concentration of 50 mg L^−1^. Ten milliliters of the U(VI) solution was added to sterile anaerobic bottles, which were then purged with nitrogen for 40 min using a nitrogen generator to remove oxygen. Then, the washed cell suspensions were redissolved to an optical density 600 nm (OD_600_) of 0.1. Cultures were incubated at 30 °C and 180 rpm under anaerobic conditions. Samples were collected at 0, 6, 12, 24, and 48 h. During sampling, the bottles were placed in an anaerobic chamber, and the suspensions were filtered through a 0.22 μm filter and centrifuged at 12,000 rpm for 10 min at 4 °C. The concentration of U(VI) was measured using inductively coupled plasma optical emission spectrometry (ICP–OES).

### 2.3 Electrochemical characteristics of *SynComs*

The microbial electrochemical cell employed in this study is illustrated in Supplementary Fig. S1. The setup consisted of a three-electrode, single-chamber reaction cell with an Ag/AgCl reference electrode, a platinum wire counter electrode, and an indium tin oxide (ITO) working electrode. The initial cell suspension (6 mL, OD_600_ = 0.1) was introduced into the chamber, and anaerobic conditions were established by purging with nitrogen gas for over 40 min. The suspensions were electrochemically incubated for 24 h at a poised potential of 0.40 V vs. standard hydrogen electrode (SHE) to form the biofilm on an ITO electrode. Carbonyl cyanide 3-chlorophenylhydrazone (CCCP; Aladdin, China), pyocyanin (PYO; Aladdin, China), phenazine-1-carboxylic acid (PCA; Aladdin, China), 1-hydroxy-phenazine (1-OH-PHZ; Aladdin, China), and deuterium oxide (D_2_O; Aladdin, China) were added upon or after the addition of cells. Cyclic voltammetry (CV) was conducted within a potential range from −0.60 to 0.25 V at a scan rate of 10 mV s^−1^. Square wave voltammetry (SWV) parameters were set with a potential increment of 0.004 V, an amplitude of 0.025 V, and a frequency of 15 Hz.

The conduction current (*I*_cond_, equation (1)) was obtained through electrochemical gating experiments, in which the ITO working electrode was replaced by an ITO interdigitated electrode (IDE) featuring a 10 μm gap between the source and drain electrodes.

(1) $$I_{\text{cond}}=\frac{I_{\text{drain}}-{\;I}_{\text{source}}}{2}$$

In this equation, *I*_drain_ (μA) is the drain current, derived from the CV curve of the IDE at a higher potential, and *I*_source_ (μA), the source current, originating from the CV curve of the IDE at a lower potential. The scan offset potential between the drain and source electrodes was set at 0.02 V with a scan rate of 1 mV s^−1^. After at least ten scans, the system yielded stable CVs [18].

Using a protein–ligand binding model, we compared the dissociation constant (*K*_d_) of flavin, outer membrane *C*-type cytochrome (OM *C*-Cyts), and flavin-bound *C*-Cyts using equations (2), (3) [19]:

(2) $$\alpha =\frac{I_{p2}}{I_{p1}}$$

(3) $$K_{d}=\frac{\left(\alpha -1\right)L_{1}}{1-\frac{\alpha L_{1}}{L_{2}}}$$

where *I*_p1_ (μA) and *I*_p2_ (μA) represent the peak currents measured by SWV at flavin concentrations *L*_1_ (μM) and *L*_2_ (μM), respectively. The ratio of these currents yields the enhancement factor (*α*), reflects the extent of protein–ligand association.

### 2.4 Sampling and quantification of metabolites, NAD^+^/NADH, and ATP

To monitor dynamic changes in metabolites during U(VI) reduction and electrochemical processes, we collected 500 μL of culture at 0, 6, 16, 18, and 24 h. After centrifugation at 6000 rpm for 3 min, the supernatants were pretreated with hydrogen-type guard columns and appropriately diluted. We analyzed organic acids (lactate, formate, and acetate) using ion chromatography (883, Metrohm, Switzerland) equipped with a Metrosep Organic Acids 250/7.8 column. The eluent was 0.5 mmol L^−1^ sulfuric acid, the suppressor regenerant solution was 100 mmol L^−1^ lithium chloride (LiCl), and ultrapure water (18.2 MΩ cm) served as the suppressor rinse solution. Detection was performed via conductivity.

To evaluate cellular redox status and energy metabolism, we processed bacterial pellets from the 24-h time point using an NAD^+^/NADH Assay Kit with WST-8 (Beyotime, China) and an ATP Assay Kit (Beyotime, China). For NAD^+^/NADH quantification, the cells were lysed and deproteinized, followed by separate derivatization reactions for NAD^+^ and NADH. WST-8 was then added as a colorimetric probe, and absorbance was measured at 450 nm after incubation, according to the manufacturer's protocol. ATP concentrations were determined based on a luminescent reaction system and quantified using a standard curve. All absorbance and luminescence signals were measured using a SpectraMax iD5 multi-mode microplate reader (Molecular Devices, United States).

### 2.5 Microscopy analysis to visualize the *SynComs*

The fluorescent dyes were diluted according to the manufacturer's instructions and stored until use. SYTO™ 60 (10 μM; Thermo Fisher Scientific) and TOTO-1 (10 μM; Thermo Fisher Scientific) dyes were mixed in a 1:1 ratio (2 mL total). After biofilms formed on the ITO glass, they were gently rinsed twice with buffer solution while preserving the biofilm. We then incubated 200 μL of dye on the biofilm at 37 °C for 1 h before aspirating the excess. The green fluorescence of TOTO-1 was observed at a wavelength of 488 nm, and the red fluorescence of SYTO™ 60 was observed at 638 nm using a Leica microscope. For transmission electron microscopy (TEM), we collected cells by centrifuging 15 mL of cell cultures at 6400 rpm for 5 min at 37 °C. The cells were then washed and fixed overnight at 4 °C in a mixed solution of 4 % paraformaldehyde and 2.5 % glutaraldehyde. After that, the cells were washed with HEPES (50 mM, pH 7.4) for TEM observation. X-ray photoelectron spectroscopy (XPS; Thermo Scientific K-Alpha, United States) spectra in the U 4f region were deconvoluted using CasaXPS software (version 2.3.13). Binding energies were calibrated using charge correction at 284.6 eV.

### 2.6 Double-stranded eDNA synthesis for *S*.MR-1 EET

Two strands (50 μM) were mixed in 200 μL of phosphate-buffered saline (PBS; pH 7.0, 5 mM NaH_2_PO_4_, 50 mM NaCl) within an anaerobic glove box. Subsequently, annealing was performed on a PCR machine by heating to 90 °C within 1 min, followed by slow cooling over a period of 90 min. DNA-modified electrodes were prepared using methods described in the literature [13]. A three-electrode cell structure was then constructed. For electrochemical measurements, one group consisted of DNA-modified electrodes immersed in a DM-L solution without bacteria, and the CV curves were measured after connecting them to an electrochemical workstation. In parallel, *S*.MR-1 (OD_600_ = 2.0) was added to the wells containing gold electrodes under anaerobic conditions. Control experiments used unmodified gold electrodes, which were sealed and removed from the chamber prior to CV analysis. After measurements, all electrodes were rinsed once with buffer solution (pH 7, 5 mM NaH_2_PO_4_, 50 mM NaCl).

### 2.7 Molecular dynamics simulation of cytochromes with phenazines

Molecular simulations were conducted using GROMACS software. The structures of the protein and small molecules are depicted in the Supplementary Materials. Docking files for the protein and ligand small molecules were imported into CB-Dock 2, followed by hydrogenation and charge-state visualization with Chimera. Molecular dynamics simulations were performed at a constant temperature of 300 K for 5 ns, with trajectories recorded every 10 ps. Finally, the binding energies were calculated using the molecular mechanics poisson–boltzmann surface area method.

### 2.8 Gene expression-related EET examined using QPCR

Samples used in the qPCR analysis were derived from *S*.MR-1 to quantify the expression levels of key EET-related genes affected by the *SynComs*. The samples were then immediately snap-frozen in liquid nitrogen and stored at −80 °C. RNA was extracted (Bacterial RNA extraction kit, R403, version 22.10) and its integrity verified by agarose gel electrophoresis; concentration and purity were measured using a micro-ultraviolet-visible spectrophotometer. Reverse transcription was performed using the HiScript IV 1st Strand cDNA Synthesis Kit (+gDNA wiper). RT–qPCR was performed using a ChamQ Universal SYBR qPCR Master Mix on a real-time fluorescent quantitative PCR analyzer (qTOWER3G, JENA, Germany). The expression of mRNA was normalized using 16S rRNA (primers listed in Supplementary Table S1). The relative mRNA levels were detected using the 2^−*ΔΔCt*^ method. All experiments were repeated three times.

## 3 Results

### 3.1 Strain isolation, U(VI) reduction, and metabolic interactions in synthetic microbial communities

We mixed 5 g of soil with 50 mL of ultrapure water to prepare a leachate, then mixed 250 μL of the leachate with 6 mL of *S.*MR-1 (OD_600_ = 0.1), and electrically precultured it at 0.2 V vs. saturated Ag/AgCl (indium tin oxide working electrode, platinum wire counter electrode) to selectively enrich electroactive microorganisms. After a two-day current increase was observed, cell suspensions were plated on an LB agar petri dish. A single green-pigmented colony was separated and named *Pseudomonas aeruginosa* LXZ1 (*P.*LXZ1) (Supplementary Fig. S1). Phylogenetic analysis showed that the 16S rDNA sequence of strain *P.* LXZ1 (BioSample accession: SAMN39097935) shared 92 % similarity with the whole-genome sequence of *Pseudomonas aeruginosa* DSM 50071 (Supplementary Fig. S2). The *P*. LXZ1 genome comprised 6,446,933 bp with 6077 predicted genes, totaling 5.74 Mbp, with an average gene length of 944.61 bp and a GC content of 67.06 % (Supplementary Table S2).

**Fig. 1:**
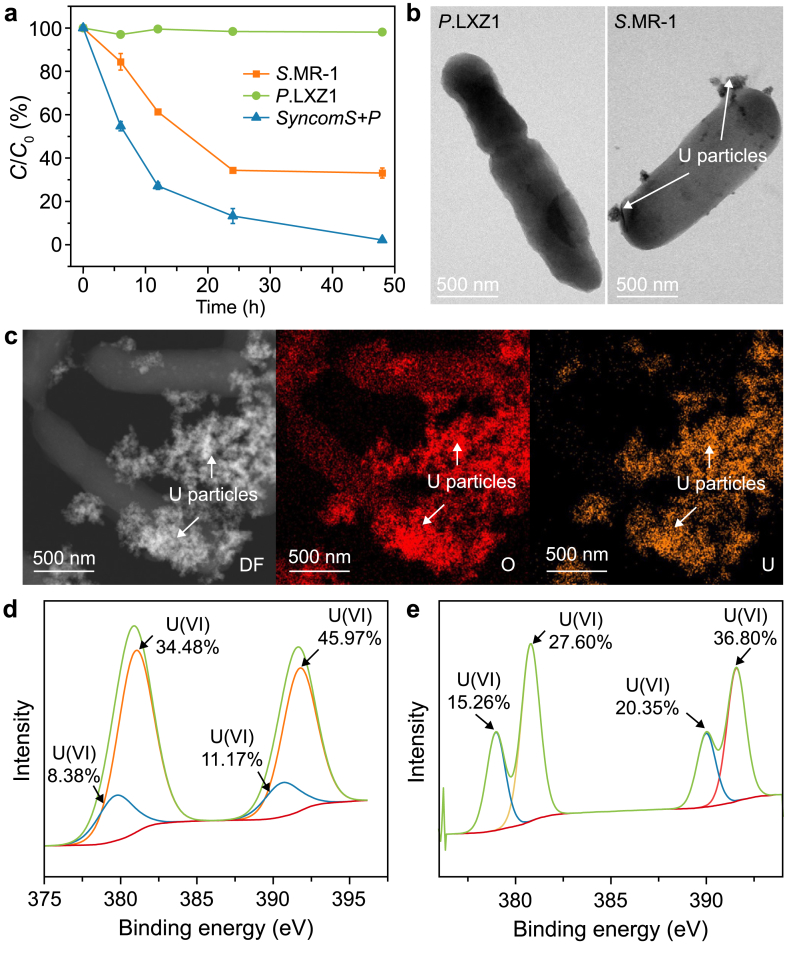
**a**, Time-resolved removal of soluble U(VI) (50 mg L^−1^) by *Shewanella oneidensis* MR-1 (*S*.MR-1), *Pseudomonas aeruginosa* LXZ1 (*P.*LXZ1), and *syncomS + P* (*S*.MR-1 + *P.*LXZ1). **b**, Transmission electron microscopy (TEM) image showing extracellular and cell-surface-associated uranium nanoparticles formed by *P.*LXZ1 and *S*.MR-1. Samples were taken after 48 h' uranium (U) removal. **c**, TEM coupled with energy-dispersive X-ray spectroscopy elemental mapping confirming U-rich deposits in the *syncomS + P* consortium, with U signals (orange) colocalized with cellular structures. **d**–**e**, XPS spectra of *S*.MR-1 (**d**) and *syncomS + P* (**e**) in the U 4f region deconvolved into U(IV) and U(VI) by using CasaXPS (v2.3.13). The fitted spectrum (yellow curve) was baseline-corrected and deconvoluted into U(IV) (blue curve) and U(VI) (green curve) components, with C 1s at 284.8 eV as the reference. The percentage reveals the coexistence of multiple uranium valence states in the sample. Data are presented as mean ± standard deviation (*n* = 3).

Subsequently, we studied the reduction rate of U(VI) via S.MR-1, *P.*LXZ1, and *SynComs S*.MR-1+*P.*LXZ1 (*syncomS + P*). The *SynComs* reduced 75 % of the U(VI) (50 mg L^−1^) within 12 h (Fig. 1a), compared with only 42 % by S.MR-1 alone. After 48 h, the *syncomS + P* had almost completely reduced the U(VI), whereas *S*.MR-1 alone achieved 60 % reduction, indicating that co-culture significantly promoted the reduction of U(VI). Unexpectedly, *P*.LXZ1 was unable to reduce the U(VI), as no U(IV) particles were observed in TEM images (Fig. 1b). XPS further confirmed only a minor adsorption of U(VI) on the surface of the *P.*LXZ1 (Supplementary Fig. S3). In contrast, both *S*.MR-1 alone and *syncomS + P* were able to remove U(VI) extracellularly by EET and form 50 nm ultra-fine particles surrounding the cell body, as observed with energy dispersive X-ray spectroscopy (EDS) mapping (Fig. 1b and c).

Notably, the *syncomS + P* produced substantially more U particles than *S*.MR-1 alone of *S*.MR-1 (Fig. 1d), consistent with the higher removal rate observed (Fig. 1b), indicating that our synthetic community accelerated the U(VI) reduction. To confirm reduction rather than adsorption, we measured the uranium valence state by XPS. We found that the proportion of U(IV) in the combination *syncomS + P* was much higher than that in *S*.MR-1, suggesting that co-culture promoted U(VI) reduction and particulate uranium formation. Overall, the efficacy of *syncomS + P* cannot be explained by adsorption or by a simple additive effect of the two strains; instead, *P.*LXZ1 appears to stimulate S.MR-1 activity, likely by enhancing metabolism and promoting electron transfer.

**Fig. 2:**
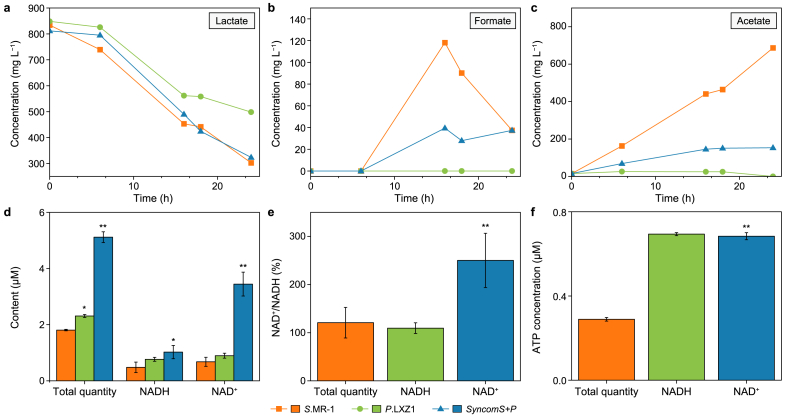
**a**–**c**, Concentrations of lactate (**a**), acetate (**b**), and formate (**c**) monitored over time under uranium-reducing conditions. **d**–**f**, The whole cell NADH and NAD^+^ contents (**d**), NAD^+^/NADH ratio (**e**), and ATP concentration (**f**) measured after 48 h' uranium removal. ∗*p* < 0.05, ∗∗*p* < 0.01. Data are presented as mean ± standard deviation (*n* = 3).

We quantified the differences in metabolites during U(IV) reduction. Within 24 h, lactate concentration decreased from 800 mg L^−1^ to 288 mg L^−1^ (*S*.MR-1), 472 mg L^−1^ (*P*.LXZ1), and 320 mg L^−1^ (*syncomS + P*) (Fig. 2a), accompanied by the accumulation of the intermediate product formate and subsequently acetate., *P*.LXZ1 largely reduced the formate and acetate accumulation compared to *S*.MR-1 (Fig. 2b and c), indicating that *P*.LXZ1 utilized formate and acetate produced by S.MR-1, thereby forming a complementary product in metabolism in *syncomS + P*.

Compared with *P*.LXZ1 and *S*.MR-1 monocultures, more aggregates appeared in *syncomS + P* (Supplementary Fig. S4a–c). TEM further revealed tight cell-to-cell binding between *S*.MR-1 and *P*. LXZ1 on a single-cell level (Supplementary Fig. S4d), which benefited the exchange of metabolic complementation. Considering that substrate metabolism is directly linked to energy and electron flow, we quantified NADH, a typical carrier of intracellular electrons. The total amount of NADH + NAD^+^ was significantly higher in *syncomS + P* than in either *S.*MR-1 or *P.*LXZ1 (Fig. 2d). Both NAD^+^ levels and NAD^+^/NADH ratios were markedly higher than those in *S*.MR-1 or *P*.LXZ1 (Fig. 2e), suggesting a faster electronic channel in *syncomS + P* for reducing U(VI). ATP concentrations in *syncomS + P* were exceeded those of *S*.MR-1 but were comparable to *P*.LXZ1, suggesting a remodeling of metabolic pathways with ATP synthesis (Fig. 2f).

### 3.2 Electrochemical characterization of EET and eDNA-Mediated enhancement in *SyncomS + P* consortia

**Fig. 3:**
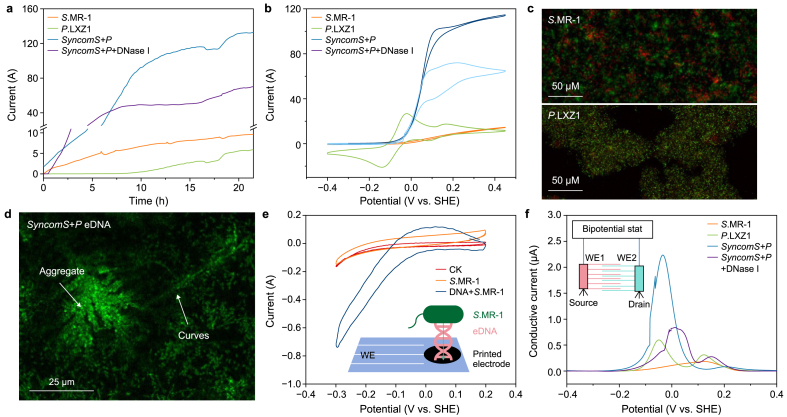
**a**–**b,** Single-potential amperograms (**a**) and cyclic voltammetry (CV; **b**) plots of *S.*MR-1, *P.* LXZ1, *syncomS + P*, and *syncomS + P* + DNase I at 0.20 V versus the saturated Ag/AgCl reference electrode at 30 °C. **c**–**d**, The fluorescent images of *S.*MR-1 and *P.* LXZ1 biofilm stained by Syto™ 60 and TOTO-1 (**c**) and *syncomS + P* (**d**). **e**, CV plots of *S.*MR-1, bare gold, and *S.*MR-1+DNA-modified printed electrodes (scan rate: 1 mV s^−1^). Control Check (CK) represents a bare electrode without any added substances. **f**, Conductive currents of *S.*MR-1, *P.* LXZ1, *syncomS + P* biofilms, and *syncomS + P* + DNase I (offset potential: 20 mV; scan rate: 1 mV s^−1^). WE1 and WE2 denote working electrode 1 and electrode 2. Data are presented as mean ± standard deviation (*n* = 3).

Given that *syncomS + P* reduces U extracellularly via EET [20,21], we established a three-electrode electrochemical system to monitor the EET processes. *P*. LXZ1 presented the lowest current output (Fig. 3a), whereas the *syncomS + P* generated currents more than 100 μA higher than either monoculture. Since these current differences closely paralleled uranium reduction rates, we used the three-electrode system to explore how the *syncomS + P* accelerates U reduction through EET.

Slow-scan CV (1 mV s^−1^) further confirmed the advantages of the *syncomS + P* consortium. For *S*.MR-1 alone, the oxidative onset potential was −100 mV, with the sigmoid current profile increasing from 1 μA to approximately 10 μA (Fig. 3b). The profile shape of *syncomS + P* was similar to *S.*MR-1, but the current plate reached 120 μA and displayed a more negative onset potential (−200 mV), leading to a 12-fold higher U reduction rate in a larger potential window. Nevertheless, the S-shaped curves observed in slow-scan CV for both *syncomS + P* and *S.*MR-1 in turnover conditions demonstrated that the electrons were sufficient to support EET. In contrast, a different shape was observed for the *P.* LXZ1 CV plot, with two large pairs of reversible peaks at −80 and 120 mV. The slow-scan CV curve of *P.* LXZ1 gradually decreased along with the positive scan potential, indicating that the electrons generated by *P.* LXZ1's metabolic processes were relatively low and insufficient to sustain the EET, leading to its inability to reduce U (Fig. 1b).

Building on the findings of Saunders et al. that conductive eDNA promoted EET inside *P*. *aeruginosa* PA14 biofilm [13], the observation of *S*.MR-1's EET promotion by *P.* LXZ1 could be ascribed to the eDNA. Therefore, we stained the biofilm with TOTO-1 (green fluorescence for eDNA) and Syto™ 60 (red fluorescence) to characterize the presence of eDNA in the biofilm. *P.* LXZ1 biofilms possessed stronger intensity than those of *S*.MR-1 (Fig. 3c and Supplementary Fig. S5, consistent with higher eDNA content. *SyncomS + P* exhibited stronger fluorescent intensity than both *P*. LXZ1 and *S*.MR-1, accompanied by the formation of 25 μm aggregates and long nanowires, suggesting that the conductive eDNA was attached to the surface of cells, which was conducive to EET.

To elucidate the key role of eDNA in *syncomS + P* biofilms, we digested eDNA using DNase I (Fig. 3a). Initially, a slight effect of DNase I addition was observed. However, the difference in current between *syncomS + P* and *S*.MR-1 enlargement after 5 h indicates biofilm formation caused by the retardation release of the eDNA. A sharp decrease of 44 % in current output was observed after DNase I was added, corresponding to a decrease in CV to 60 μA (Fig. 3a; Supplementary Fig. S6). Given that a higher current was observed at 5 h in *syncomS + P* than *S*.MR-1 alone, the DNase I showed no effect on *syncomS + P*'s metabolism in relation to electron transfer inside the cell body, strongly supporting the idea that eDNA promotes *S*.MR-1 EET.

To further verify that *S*.MR-1 used eDNA to enhance EET, we annealed a short complementary double-strained DNA containing thiol groups (Supplementary Table S3) and modified it on the gold electrode (Supplementary Fig. S7a and b) [13]. The CV plot of *S*.MR-1 on the modified gold electrode was significantly higher than the redox peak of *S*.MR-1 on the ordinary electrode (Fig. 3e), indicating that eDNA in the aggregates and long-distance nanowires directly accelerated the electron transfer of *S*.MR-1.

The above electrochemical tests demonstrated the enhancement of *S*.MR-1 EET via eDNA on the interface of the three-electrode system. To study how eDNA enhanced conductivity inside the aggregate, we replaced the ITO glass with a 10-μm-gap interdigitated electrode and measured the current difference between the electrodes at an offset potential of 20 mV (Supplementary Fig. S8) [18]. The conduction currents of *S*.MR-1 and *P*. LXZ1 were 0.10 and 1.27 μA, respectively, which is consistent with previous reports (Fig. 3f). The conduction current of *syncomS + P* was 4.36 μA, exceeding the sum of *S*.MR-1 and *P*. LXZ1 (Fig. 3f). After DNase I addition, the conduction current of *syncomS + P* dropped to 1.50 μA. This result highlights the critical role of eDNA in enhancing the EET inside the whole biofilm by promoting mixed-bacteria biofilm formation and facilitating electron transfer.

### 3.3 High pyocyanin producing strain *P.*LXZ1 mediated EET enhancement in *SyncomS + P* consortia

In addition to the eDNA, *Pseudomonas aeruginosa* strains can secrete a variety of redox–active phenazines, such as phenazine-1-carboxylic acid (PCA) and pyocyanin (PYO), for *Shewanella* to reduce electron acceptors (Supplementary Fig. S9a). Recent studies have also found that PYO binds to eDNA, facilitating rapid electron transfer inside biofilm [12,13]. Thus, we speculated that *P.* LXZ1's superior EET promotion was also related to phenazines. We predicted secondary metabolite gene clusters using anti-SMASH (version 7.1.0) [22], and the results showed that the *P.* LXZ1 genome contained multiple secondary metabolite gene clusters, including phenazines, thiopeptide antibiotics, and 19 other gene clusters (Supplementary Table S4). Among them, gene cluster 13 showed 100 % homology with PYO.

**Fig. 4:**
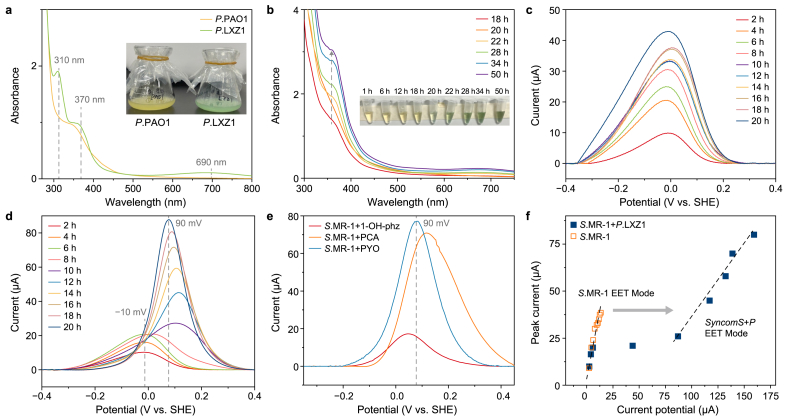
**a**, Ultraviolet visible absorption spectra (UV–vis) spectra of the *P.* PAO1 and *P.* LXZ1 supernatants after 24 h' cultivation in the LB medium at 30 °C. The inset panel presented the image of the *P.* PAO1 and *P.* LXZ1 cell suspension in an Erlenmeyer flask. **b**, Variation in the time-dependent UV–vis of *P.* LXZ1 cell suspension. The inset panel presented the image of the *P.* LXZ1 culture supernatants collected at different time points within 24 h. **c**–**e**, Variation in the time-dependent square wave voltammograms of *S*.MR-1 (**c**), *syncomS + P* (**d**), and *S*.MR-1 with different phenazine PYO, PCA, and 1-OH-phz at 20 h (**e**). **f**, Plot of current output against the square wave voltammetry peak current of *S*.MR-1 and *syncomS + P*. Gray dotted lines highlight the spatial alignment of peak positions. Gray arrows indicate the shift in the electron transfer pathway from the conventional extracellular electron transfer (EET) mode.

The supernatant of the *P.* LXZ1 gradually turned deep blue–green (Fig. 4a and b), while *P*. *aeruginosa* model strain PAO1 retained the light-yellow of the LB medium and exhibited almost no color change over 48 h. With regard to the identical growth curves of both strains (Supplementary Fig. S9b), this color alteration resulted from *P.* LXZ1's stronger ability to secrete phenazine than *P.* PAO1. We then categorized the phenazine biosynthetic pathway genes between *P.* LXZ1 and *P.* PAO1 into three clusters for analysis based on their positional distribution: *phzH* (NC_002516.2:66303–68135), *phzA2-E2* (NC_002516.2:2070685–2075438), and *phzM*, *A1-E1* (NC_002516.2:4713796–4718543). Multiple mutation sites were identified across *phzA1-G1* and *A2-G2* (Supplementary Fig. S10a–c), resulting in 115 codon mutations in the *phzA1B1* region and 116 codon mutations in the *phzA2B2* region (Supplementary Fig. S10d), which ultimately led to a significant increase in the rate of phenazine biosynthesis in *P.* LXZ1.

Distinct absorption peaks at 310, 370, and 690 nm in the ultraviolet–visible spectra featured the characterization of PYO [23], showing remarkable differences from those of PCA and 1-OH-phz. We found that U removal by *P.*PAO1+*S*.MR-1 was significantly lower than that of *P*.LXZ1+*S*.MR-1, corresponding to its lower EET rate (Supplementary Fig. S11), indicating that a high PYO-producing strain *P.*LXZ1 would lead to EET enhancement in *syncomS + P* consortia.

To understand the dynamic role of PYO in electron transport, we performed chronoamperometry while recording SWV every 2 h. The SWV peak of *S.*MR-1 increased over time (Fig. 4c), while the potential was maintained between −10 and 0 mV (Fig. 4d), which was consistent with the waveform of *syncomS + P* in the first 2–6 h. However, at 8–20 h, the peak at −10 mV decreased, accompanied by a shift to an unknown peak at approximately 90 mV and increasing over time. The peak profile was different from that of *P.* LXZ1 alone: Two distinct small peaks at −50 mV and 120 mV were observed (Supplementary Fig. S12).

To confirm whether the unknown peak was caused by PYO, we supplemented S.MR-1 with 200 μM PCA, 1-OH-phz, and PYO, compounds usually secreted by *Pseudomonas* sp. PYO produced strong current enhancement (Supplementary Fig. S13), and the single SWV peak of S.MR-1+PYO matched the shape and position of the unknown peak (Fig. 4e). With regard to the distinct shapes and positions of the SWV peaks of *S*.MR-1+1-OH-phz and *S*.MR-1+PCA, it was evident that the PYO secreted by *P.* LXZ1, rather than PCA or 1-OH-phz, dominated the enhancement of EET. We next correlated SWV peaks with the current output (Fig. 4f). During the first 6 h, the slope of the *syncomS + P* consortia resembled that of *S*.MR-1, indicating reliance on the OmcA/MtrC pathway. After that, the slope shifted significantly, indicating a remarkable increase in current output that differed from *P.* LXZ1 alone (Supplementary Fig. S14). Therefore, the large increase in *syncomS + P*'s EET was not caused by the direct PYO redox activity.

### 3.4 Pyocyanin-OmcA complex mediated U(IV) reduction enhancement in *SyncomS + P*

**Fig. 5:**
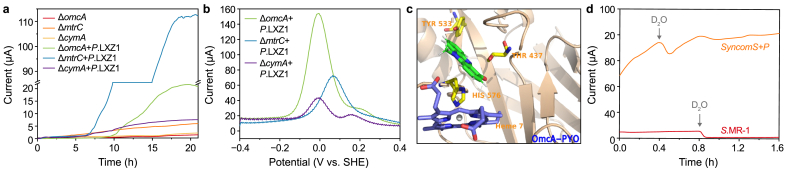
**a**, Single-potential amperograms of Δ*omcA* + *P.*LXZ1, Δ*mtrC* + *P.*LXZ1, Δ*cymA* + *P.*LXZ1, Δ*omcA*, Δ*mtrC*, and Δ*cymA* (0.20 V versus saturated Ag/AgCl at 30 °C). **b**, Square wave voltammograms of Δ*omcA* + *P.*LXZ1, Δ*mtrC* + *P.*LXZ1, and Δ*cymA* + *P.*LXZ1 after the single-potential amperometry measurement. **c**, Molecular dynamics simulations between *C*-type cytochrome OmcA of *S*.MR-1 and PYO. **d**, Single-potential amperograms of *S.*MR-1 and *syncomS + P* before and after adding 60 μL deuterium oxide (D_2_O) at 0.2 V versus saturated Ag/AgCl at 30 °C.

Recent studies have discovered that PCA can bind to *C*-type cytochromes and promote the bilateral electron transfer of *S*.MR-1 [24]. Considering that the addition of CCCP completely suppressed the EET rate, associated with a drop in the SWV peak (Supplementary Fig. S15), we infer that *C*-type cytochromes interacted with PYO during electron flow [16,25]. Herein, PYO secreted by *P.* LXZ1 bound to *S*.MR-1, causing the peak potentials to shift. The loss of the three proteins OmcA/MtrC/CymA led to a significant decrease in the current of *S*.MR-1 (Fig. 5a). However, the current output of the Δ*mtrC* + *P*.LXZ1 combination restored to the level of *syncomS + P*, whereas Δ*omcA* + *P*.LXZ1 only partially recovered, indicating that OmcA specifically binds PYO. In fact, the U(VI) reduction rate of the Δ*omcA* + *P*.LXZ1 co-culture was only 10 % after 6 h, which was much lower than the 75 % of *syncomS + P* (Supplementary Fig. S16), underscoring the role of OmcA–PYO interactions in enhancing U(VI) reduction.

We further performed SWV scanning on the co-culture system of mutants (Δ*omcA,* Δ*mtrC,* and Δ*cymA*) with *P.*LXZ1. The SWV peak of Δ*mtrC* + *P.*LXZ1 appeared at 90 mV, identical to *syncomS + P*, whereas Δ*omcA* + *P*.LXZ1 showed a −50 mV peak resembling *P*.LXZ1 alone. These results indicate a stable interaction between OmcA and PYO, but only a weak interaction between MtrC and PYO (Fig. 5b) [26], consistent with the current output (Fig. 5a). Notably, the loss of CymA restored the peak of Δ*cymA* + *P*.LXZ1 to *P*. LXZ1, indicating that the binding peak needed electrons from CymA to reduce MtrC/OmcA, which we demonstrated in a previous study [16].

We employed molecular dynamics (MD) simulations to compare the binding affinities of OmcA and MtrC with PYO. Initially, docking site predictions were performed for the proteins and ligand molecules (Supplementary Fig. S17), yielding 20 docking results. Based on literature and scoring evaluations, heme 7 in OmcA/MtrC was selected as the primary docking site for PYO, PCA, and RF (Fig. 5c; Supplementary Fig. S18). Root-mean-square deviation (RMSD) analyses confirmed that the systems reached stable conformations (Supplementary Fig. S19), after which we calculated the average binding energy during the 10–20 ns simulation window. The binding energy of OmcA–PYO was −105.18 ± 1.27 kJ mol^−1^ (Table 1), significantly lower than that of MtrC–PYO (−34.67 ± 1.53 kJ mol^−1^), indicating a much stronger binding affinity between OmcA and PYO compared to MtrC–PYO. This finding is consistent with the electrochemical behavior of the mutant strains. The van der Waals interaction energy for OmcA–PYO (−121.34 ± 1.07 kJ mol^−1^) was lower than that for MtrC–PYO (−71 kJ mol^−1^), further confirming stronger OmcA–PYO binding. Additional MD simulations showed that both PCA and PYO bound OmcA with significantly lower energies than RF, despite previous reports of specific noncovalent interactions between OmcA and RF.

**Table 1:** The binding energy of the heme protein and molecules.

Energy components	Binding energy (kJ mol^−1^)			
	OmcA–PYO	MtrC–PYO	OmcA–RF	OmcA–PCA
Van der Waal	−121.34 ± 1.07	−71.86 ± 0.68	−90.42 ± 1.82	−93.49 ± 0.97
Electrostatic	−3.63 ± 0.83	−27.58 ± 0.43	−11.67 ± 1.40	−13.79 ± 0.37
Polar solvation	30.52 ± 0.94	72.33 ± 1.99	75.09 ± 3.38	61.69 ± 1.19
SASA	−10.70 ± 0.09	−7.58 ± 0.09	−11.60 ± 0.20	−9.55 ± 0.09
Binding	−105.18 ± 1.27	−34.66 ± 1.53	−38.50 ± 2.06	−55.09 ± 1.30

Note: Van der Waals and Electrostatic terms were obtained from molecular mechanics energy calculations. Polar solvation was estimated by solving the Poisson–Boltzmann equation. Solvent accessible surface area (SASA) represents the nonpolar solvation energy approximated by the solvent-accessible surface area model. The total binding energy (Binding) is the sum of these contributions. PYO, pyocyanin; PCA, phenazine-1-carboxylic acid; RF, riboflavin.

To validate our simulations, we used SWV to quantitatively determine the binding constants of PYO/RF with *S.*MR-1 and *syncomS + P* (Supplementary Fig. S20). The dissociation constants (*K*_d_) were calculated using equations (2), (3), as described [27], the values were 5.04 μM (*S*.MR-1+RF), 0.69 μM (*S*.MR-1+PYO), and 0.50 μM (*syncomS + P* + PYO). *K*_d(S.MR-1+PYO)_ decreased by 4.9 times as much as *K*_d(*S.*MR-1+RF)_, confirming the significantly stronger binding affinity between PYO and OmcA compared to RF. Moreover, *K*_d(*syncomS + P*_ _+ PYO)_ was 27 % lower than *K*_d(*S*.MR-1+PYO)_, suggesting that in the synthetic community biofilm, the binding affinity was enhanced compared to the *S.*MR-1 biofilm. These results are consistent with our MD simulations, collectively showing that phenazine PYO produced by *P.* LXZ1 interacts more strongly with the *C*-type cytochrome OmcA of *S.*MR-1 than with other cofactors, enhancing extracellular electron transfer efficiency.

As reported by Okamoto et al. [28], the EET process in *S*.MR-1 is limited by proton transport, which generates a charge imbalance and constrains both electron transfer rates and U(VI) immobilization. To test this limitation, we added D_2_O. Upon the addition of 1 % D_2_O, the current rapidly decreased by 82 % (from 5.6 to 1.0 μA) without recovery (Fig. 5d), corresponding to a strong deuterium kinetic isotope effect (KIE) and confirming proton-coupled EET [29]. By contrast, in *syncomS + P*, the current decreased by only 12 % (75.0–64.0 μA) and fully recovered within 10 min, indicating that the *syncomS + P* markedly alleviated proton-transfer limitations.

### 3.5 Enhancement of U(IV) reduction through upregulation of EET-related metabolism pathways

**Fig. 6:**
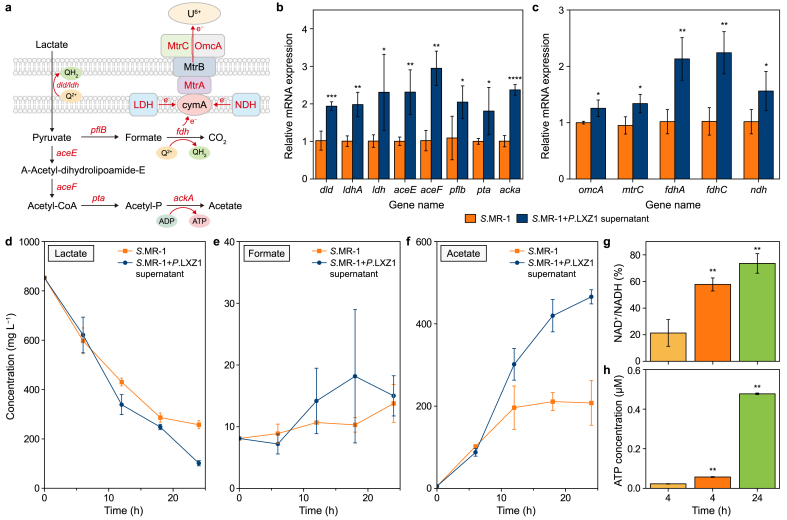
**a,** The scheme of the *S*.MR-1 metabolic pathway. ATP: adenosine triphosphate; ADP: adenosine diphosphate. **b**–**c**, Gene expression related to energy metabolism (**b**) and electron transfer (**c**) in *S*.MR-1. **d**–**f**, Alteration of lactate (**d**), formic acid (**e**), and acetate (**f**) monitored over time. **g**, NAD^+^/NADH ratio. **h**, ATP contents. ∗*p* < 0.05, ∗∗*p* < 0.01, ∗∗∗*p* < 0.001, and ∗∗∗∗*p* < 0.0001. Data are presented as mean ± standard deviation (*n* = 3).

We revealed that *P.*LXZ1 facilitated the reduction of U(VI) primarily via conductive PYO and eDNA. Since the *P.*LXZ1 influence on gene expression—particularly with regard to EET and metabolism in *S.*MR-1—was obscure, we conducted qPCR. The metabolic pathway in *S.*MR-1 is illustrated in Fig. 6a. Lactate is initially converted to pyruvate, then to acetyl-CoA, and ultimately to acetate. Notably, all genes related to the metabolic pathways were ∼2-fold upregulated (red labels; Fig. 6b), indicating that electron transfer in *S.*MR-1 is constrained by upstream metabolism [18,30]. Here, *syncomS + P* significantly improved the electron supplement to EET via the upregulation of three key genes (*ldh, ndh,* and *fdh*), alleviating the metabolic constraints of EET. Meanwhile, EET-related *C*-type cytochromes OmcA/MtrC, which serve as key proteins for uranium reduction, exhibited a moderate increase of 1.2-fold (Fig. 6c).

We also quantified the concentration changes of the key metabolic products (Fig. 6d–f). After 24 h, the lactate concentration in the *syncomS + P* supernatant group decreased to 100 mg L^−1^, significantly lower than the 250 mg L^−1^ observed in the *S.*MR-1 group, which corresponds to the increased expression of the *dld* gene. Pyruvate and acetyl-CoA were not detected due to rapid turnover, but formate accumulated, aligning with upregulation of *pflB*. After 24 h, the formate levels in *S.*MR-1 decreased, indicating its oxidation to CO_2_, which enhanced quinone production and electron transfer. In addition, *ackA*, the only ATPase functioning in the anaerobic EET process, catalyzed the conversion of acetyl phosphate to acetate while generating ATP. The *syncomS + P* supernatant significantly increased the final acetate concentration from 200 to 450 mg L^−1^ (Fig. 6f), indicating substantial ATP synthesis. Indeed, our final quantification of NADH and ATP revealed that although the total NADH + NAD ^+^ pool remained unchanged, the NAD^+^/NADH ratio increased from 20 % to 55 %, eventually reaching 70 % after 24 h (Fig. 6g), reflecting rapid electron transfer and substantial NADH consumption. ATP production also showed a significant increase, from 0.025 μM in *S.*MR-1 to 0.05 μM in *syncomS + P* supernatant, ultimately reaching 0.5 μM after 24 h (Fig. 6h). In summary, the combination of *S.*MR-1 with *P*. LXZ1 enhanced uranium reduction by accelerating upstream metabolism.

## 4 Discussion

While the model strain of DMRB, *S*.MR-1, has been studied extensively for its ability to immobilize U(VI) via EET, the reduction rate of U(VI) remains limited. We systematically compared the EET-enhancing effects of exogenous phenazines, flavins, and quinones [31] and found that phenazines exhibited a much stronger ability to enhance EET at relatively low concentrations than the other compounds [31]. Consistent with this, MD simulations and dissociation constant analyses showed that the OmcA–PYO complex had a binding energy of −105.18 ± 1.27 kJ mol^−1^, substantially lower than that of the OmcA–PCA (−55.09 ± 1.30 kJ mol^−1^) or OmcA–RF (−38.50 ± 2.06 kJ mol^−1^). The dissociation constant of PYO was only 20 % of that of RF, indicating that even a small amount of PYO can exert a significant effect on U(VI) reduction. This suggests that PYO is more advantageous in promoting EET under low-concentration or open subsurface soil and groundwater conditions, thereby reducing the metabolic burden on microbial communities to express endogenous mediators.

In this work, we isolated the strain *P.*LXZ1, significantly promoting the secretion of phenazine PYO. While PYO itself has strong redox activity and can rapidly donate electrons, Newman et al. found that PYO passed through the outer membrane and periplasm space and was mainly reduced by NDH in the inner membrane [32], corresponding to the low rate of PYO reduction inside *P.*LXZ1 cells and leading to the inability to reduce U(VI). In contrast, the extracellular OmcA of *S.*MR-1 can directly interact with PYO spatially, supporting rapid U(VI) reduction.

**Fig. 7:**
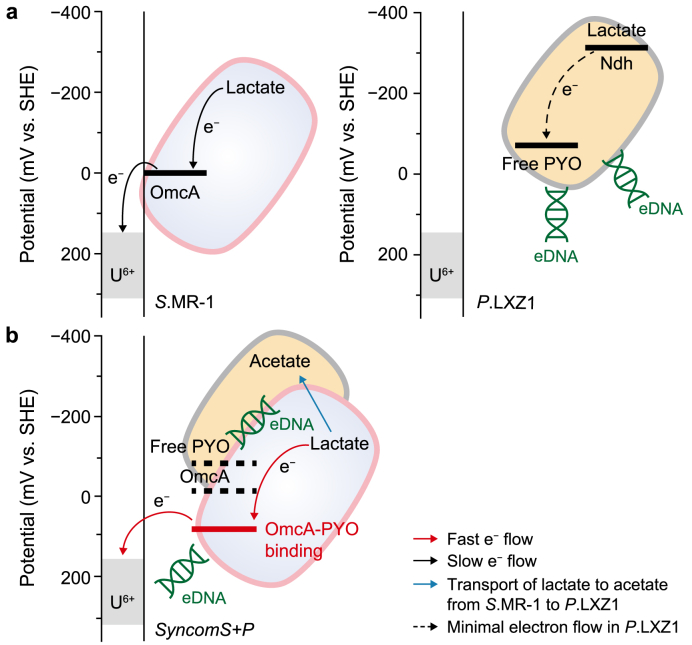
The energy scheme diagram of uranium reduction conducted by *S.*MR-1 and *P.* LXZ1 individually (**a**) or accelerated by the synthetic consortia *syncomS + P* (**b**). The red arrows indicate the rapid electron flow in *syncomS + P*, while the black arrows denote the normal electron flow in *S.*MR-1. The blue arrows represent the transport of lactate to acetate from *S.*MR-1 to *P.* LXZ1, and the black dotted arrows illustrate the minimal electron flow in *P.* LXZ1. The bold black solid and dotted lines indicate the redox potentials corresponding to the respective reactions, while the red line represents the redox potential after *syncomS + P* formation.

From an energetic perspective, electrons transfer from the high-potential OmcA (0 mV) to the lower-potential PYO (−40 mV), indicating that a significant energy barrier must be overcome, making the process thermodynamically unfavorable. Studies by Okamoto et al. have shown that the OmcA–RF complex formation shifts the peak potential of RF from a lower −240 to −145 mV [33], which remains thermodynamically disadvantageous due to the higher potential of OmcA at +50 mV. Our study, for the first time, discovered that PYO has a similar structure to RF, forming a complex with the OmcA–PYO binding complex and shifting the potential from −40 to 90–100 mV, which facilitates electron transfer from OmcA. This shifting allows electron transfer to flow in a sequence of the redox potential gradient, reversing the thermodynamically unfavorable situation of EET and increasing the EET rate (Fig. 7).

Furthermore, this study found that the eDNA secreted by *P.*LXZ1 plays a crucial role in promoting the formation of mixed biofilms and electron transfer in the synthetic microbial community. The eDNA adheres to *S*.MR-1 cells to form aggregates (Fig. 2), which facilitates the utilization of metabolic products within the microbial community in a short space, thereby enhancing the stability of the synthetic microbial community and promoting the adhesion of *S*.MR-1 to uranium. Moreover, the current drop after the addition of DNase I confirmed that removing eDNA resulted in a significant decrease in electron transfer efficiency, underscoring the crucial role of eDNA in enhancing the conductivity of biofilms. We also found that the electroactivity of *S*.MR-1+artificial synthetic eDNA was significantly higher than that of *S*.MR-1 alone (Fig. 3), demonstrating that eDNA itself can assist *S*.MR-1 in directly transferring electrons.

Although *P.*LXZ1 itself is incapable of reducing U(VI), the PYO and eDNA secreted by *P.* LXZ1 extend the electron transfer pathway, greatly promoting the U(VI)-reducing function of the core strain *S*.MR-1. This is also supported by transcriptomic and related metabolic data: *syncomS + P* promotes the expression of metabolic core modules with key genes related to energy metabolism, such as NADH and quinone production, and ATP synthesis, significantly upregulating these processes. This corresponds to a prominent increase in the NAD^+^/NADH ratio and an increase in ATP synthesis, indicating that *P.* LXZ1 effectively enhances the energy supply in the U(VI) reduction process by boosting the existing metabolic pathways of *S*.MR-1, thereby enhancing its electron transfer capability.

## 5 Conclusion

From the perspective of synthetic microbial communities, this study demonstrates an innovative approach to constructing a highly efficient collaborative system of microbial functional modules. Through metabolic complementarity and optimization of electron transfer pathways, we achieved seamless integration of the two strains’ functions, reflecting the design concept of synthetic microbial communities based on functional division of labor and collaboration. Unlike traditional single-strain electron transfer pathway optimization, this study shows the potential of exogenous molecules in regulating electron transfer in multi-strain systems. In addition, the formation and enhanced conductivity of mixed biofilms mediated by eDNA provide new insights for developing more efficient microbial biofilm systems. This strategy not only strengthens the function of biofilms but also offers broad prospects for their application. In summary, our study provides a novel tool for pollution remediation, demonstrating the superior efficiency of synthetic microbial communities in reducing U(VI) and their significant application value.

## CRediT authorship contribution statement

**Xizi Long:** Writing – review & editing, Writing – original draft, Visualization, Validation, Supervision, Methodology, Investigation, Funding acquisition, Data curation, Conceptualization. **Yuanyuan Jiang:** Writing – original draft, Visualization, Methodology, Investigation. **Zhaozhong Zhu:** Methodology, Data curation. **Yu Li:** Writing – original draft, Visualization. **Nan Hu:** Writing – original draft. **Junzhan Hong:** Methodology, Investigation. **Hui Wang:** Writing – review & editing, Writing – original draft, Conceptualization. **Fei Yang:** Writing – review & editing, Writing – original draft.

## Competing of interests

The authors declare that they have no known competing financial interests or personal relationships that could have appeared to influence the work reported in this paper.
