# Supplementary material for: Complete uranium bioreduction in 48 hours: Synergistic electron transfer in a synthetic microbial consortium
Source: Environ Sci Ecotechnol. 2025 Oct 21;28:100629. doi: 10.1016/j.ese.2025.100629 (PMC12595025; doi:10.1016/j.ese.2025.100629)
Supplement: Multimedia component 1 [file mmc1.docx]

**Supporting Information**

**Complete Uranium Bioreduction in 48 Hours: Synergistic Electron Transfer in a Synthetic Microbial Consortium**

Xizi Long^1,^**^†^**^,^*, Yuanyuan Jiang^1,†^, Zhaozhong Zhu^1^, Yu Li^2^, Nan Hu^3^, Junzhan Hong^4^, Hui Wang^2,^*, Fei Yang^1,^*

^1^The Key Laboratory of Typical Environmental Pollution and Health Hazards of Hunan Province, School of Public Health, University of South China, Hengyang 421001, China

^2^Department of Municipal and Environmental Engineering, Faculty of Water Resources and Hydroelectric Engineering, Xi’an University of Technology, Xi’an 710048, China

^3^Key Discipline Laboratory in Uranium Mining and Hydrometallurgy, University of South China, Hengyang, Hunan, 421001, China

^4^Hunan Provincial Key Laboratory of Geochemical Processes and Resource Environmental Effects, Geophysical and Geochemical Survey Institute of Hunan, Changsha, Hunan 410014, China.

**Contents Summary:**

**Table S1-S6 (page S2-S4)**

**Figures S1-S20 (page S4-S16)**

Table S1 Primers used in this research.

| Gene name | Primer F | Primer R |
| --- | --- | --- |
| *dld* | AGAATCGTGCCTGAAGTTGTTGTC | GTTCCTGCCGCCCTAAATGTG |
| *ldhA* | CTCGGTTTTGGCTGTAAAGTGATTG | GCTGGTGGCGTAAATGGTATCC |
| *ldh* | AAGATCGCCGTTCAAGGTGTTG | TCAAGTGACGCCTGATGAATATCG |
| *aceE* | CCAGAAGGTGCCGAGGAAGG | TCAAGAATAGTGCCACAACTCAGTAAC |
| *aceF* | GCGGCGGCGAAGAAGAAAG | TGCTTAGGCTTGAGTTGAACACAG |
| *pflB* | CCTCATTAACCTCTGTGGCTAAGC | GTGCGGCGACCATCTTCATC |
| *pta* | CACTACCGCCAACACCATTCG | GTCACCGTACACCAGCACTTG |
| *ackA* | CGTGATTAAAGGCATTGAAGAGTGTTC | CGGCGACTTGTGGCAGTTTAG |
| *mtrC* | TGAGCCAGCAGGTAGCATCC | GGCATGTCGGCTTCGTTAGTG |
| *omcA* | GAATGTTGAGTTGTATGCTGGTGTAG | GGTTGGTCTTGGATGTAAGCGTAG |
| *fdhA* | GCTCAGTAGGTTGTGGTGTGTATG | TAGCACAGTGACCGCCTTGG |
| *fdhC* | TATCTTTATCGTGTCGTGGATTGTCTG | TGATGTAGCCACCGACCACTAAG |
| *ndh* | TGGCTACTAAGCGAATAGTGATTGTG | ATCAATTAAGCAGACATCAACGACATC |

Table S2 General genomes feature of *Pseudomonas aeruginosa* LXZ1

| **Sample Name**  **（#）** |  |  |  |  |  | **Genome**  **Size**  **（#）** | **Total**  **Number（#）** | **Total**  **Length（bp）** | **Average**  **Length**  **（#）** | **Genome**  **Length**  **（%）** | **GC**  **Content（%）** |
| --- | --- | --- | --- | --- | --- | --- | --- | --- | --- | --- | --- |
| *P*.LXZ1 |  |  |  |  |  | 6446933 | 6077 | 5740371 | 944.61 | 89.04 | 67.06 |

Table S3 DNA sequence for modified electrodes

| Single strand (5' to 3') | Single strand (5' to 3') |
| --- | --- |
| TCGACGTCGTACTGAGC | AGCTGCAGCATGACTCG |
| 5' end modification**：**5`SH C6 | 5' end modification：5`SH C6 |

Table S4 General genomes feature of *Pseudomonas aeruginosa* LXZ1

| **Region** | **Type** | **From** | **To** | **Most similar known cluster** | **similarity** |
| --- | --- | --- | --- | --- | --- |
| Region 1 | NRP-metallophore,NRPS,phenazine | 728209 | 789638 | pyochelin | 92% |
| Region 2 | RiPP-like | 898216 | 909046 |  |  |
| Region 3 | NRPS-like | 915168 | 958143 | MA026 | 5% |
| Region 4 | hserlactone | 1663903 | 1684508 |  |  |
| Region 5 | NAGGN | 1687970 | 1702730 |  |  |
| Region 6 | NRPS | 1847431 | 1894489 | azetidomonamide A/azetidomonamide B | 100% |
| Region 7 | RiPP-like | 1923278 | 1934132 |  |  |
| Region 8 | NRPS,NRP-metallophore | 2904701 | 3026474 | Pf-5 pyoverdine | 23% |
| Region 9 | NRPS | 3079812 | 3132023 | L-2-amino-4-methoxy-trans-3-butenoic acid | 100% |
| Region 10 | hydrogen-cyanide | 3,266,637 | 3,279,547 | hydrogen cyanide | 100% |
| Region 11 | redox-cofator | 3,499,180 | 3,521,324 | lankacidin C | 13% |
| Region 12 | thiopeptide | 3,564,514 | 3,597,517 | oxalomycin B | 6% |
| Region 13 | phenazine | 3,597,885 | 3,618,936 | pyocyanine | 100% |
| Region 14 | hserlactone | 4,191,216 | 4,211,836 |  |  |
| Region 15 | NRPS-like,betalactone | 4,330,431 | 4,372,287 | pyoverdine SMX-1 | 12% |
| Region 16 | opine-like-metallophore | 5,592,658 | 5,614,747 | pseudopaline | 100% |

Table S5 Nadh dehydrogenase-related gene ID and functional description

| Gene ID (SO_RS) | (Nadh dehydrogenase)Description |
| --- | --- |
| gene-SO_RS04770 | NADH-quinone oxidoreductase subunit A |
| gene-SO_RS04765 | NADH-quinone oxidoreductase subunit B |
| gene-SO_RS04760 | NADH-quinone oxidoreductase subunit C/D |
| gene-SO_RS04755 | NADH-quinone oxidoreductase subunit NuoE |
| gene-SO_RS04750 | NADH-quinone oxidoreductase subunit NuoF |
| gene-SO_RS04745 | NADH-quinone oxidoreductase subunit NuoG |
| gene-SO_RS04740 | NADH-quinone oxidoreductase subunit NuoH |
| gene-SO_RS04735 | NADH-quinone oxidoreductase subunit NuoI |
| gene-SO_RS04730 | NADH-quinone oxidoreductase subunit J |
| gene-SO_RS04725 | NADH-quinone oxidoreductase subunit NuoK |
| gene-SO_RS04720 | NADH-quinone oxidoreductase subunit L |
| gene-SO_RS04715 | NADH-quinone oxidoreductase subunit M |
| gene-SO_RS04710 | NADH-quinone oxidoreductase subunit NuoN |

Table S6 F-type ATPase-related gene IDs and functional descriptions

| Gene ID (SO_RS) | (F-type ATPase)Description |
| --- | --- |
| gene-SO_RS22085 | F0F1 ATP synthase subunit alpha |
| gene-SO_RS22105 | F0F1 ATP synthase subunit A |
| gene-SO_RS22090 | F0F1 ATP synthase subunit delta |
| gene-SO_RS22080 | F0F1 ATP synthase subunit gamma |
| gene-SO_RS22095 | F0F1 ATP synthase subunit B |
| gene-SO_RS22100 | F0F1 ATP synthase subunit C |
| gene-SO_RS22105 | F0F1 ATP synthase subunit A |
| gene-SO_RS22070 | F0F1 ATP synthase subunit epsilon |
| gene-SO_RS08160 | decaheme c-type cytochrome MtrF |
| gene-SO_RS17100 | heme utilization protein HutZ |


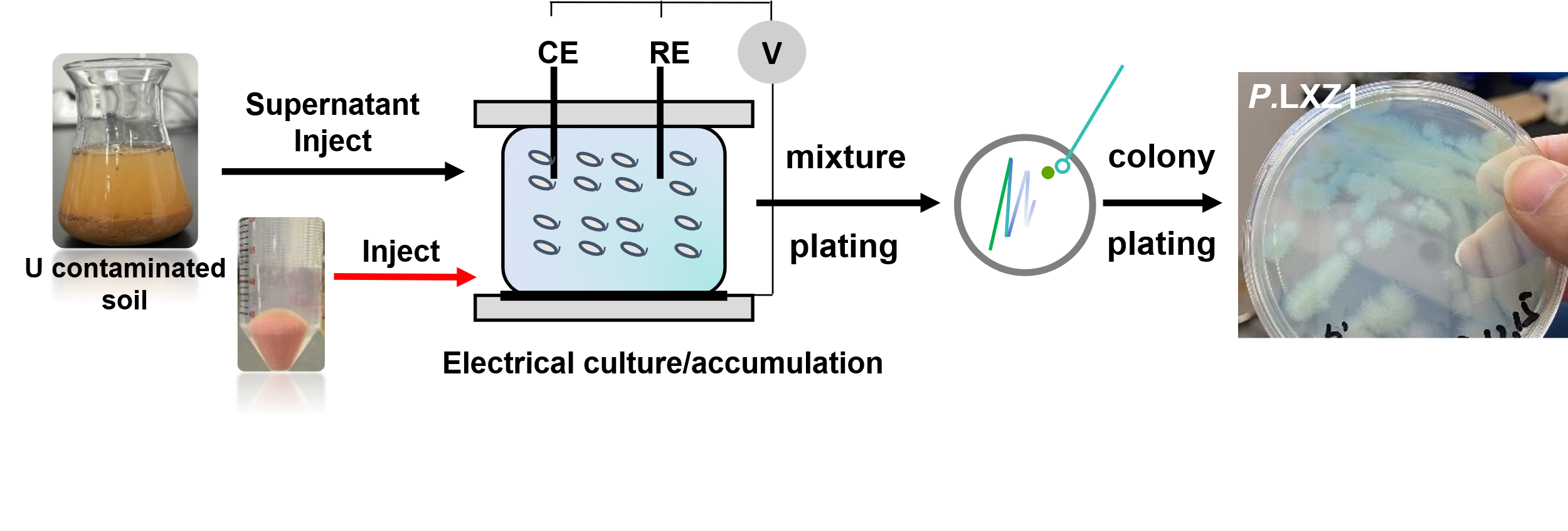


Fig S1 The scheme steps of colony isolation after electrical pre-culture incubated with soil leachate and *S*.MR-1.


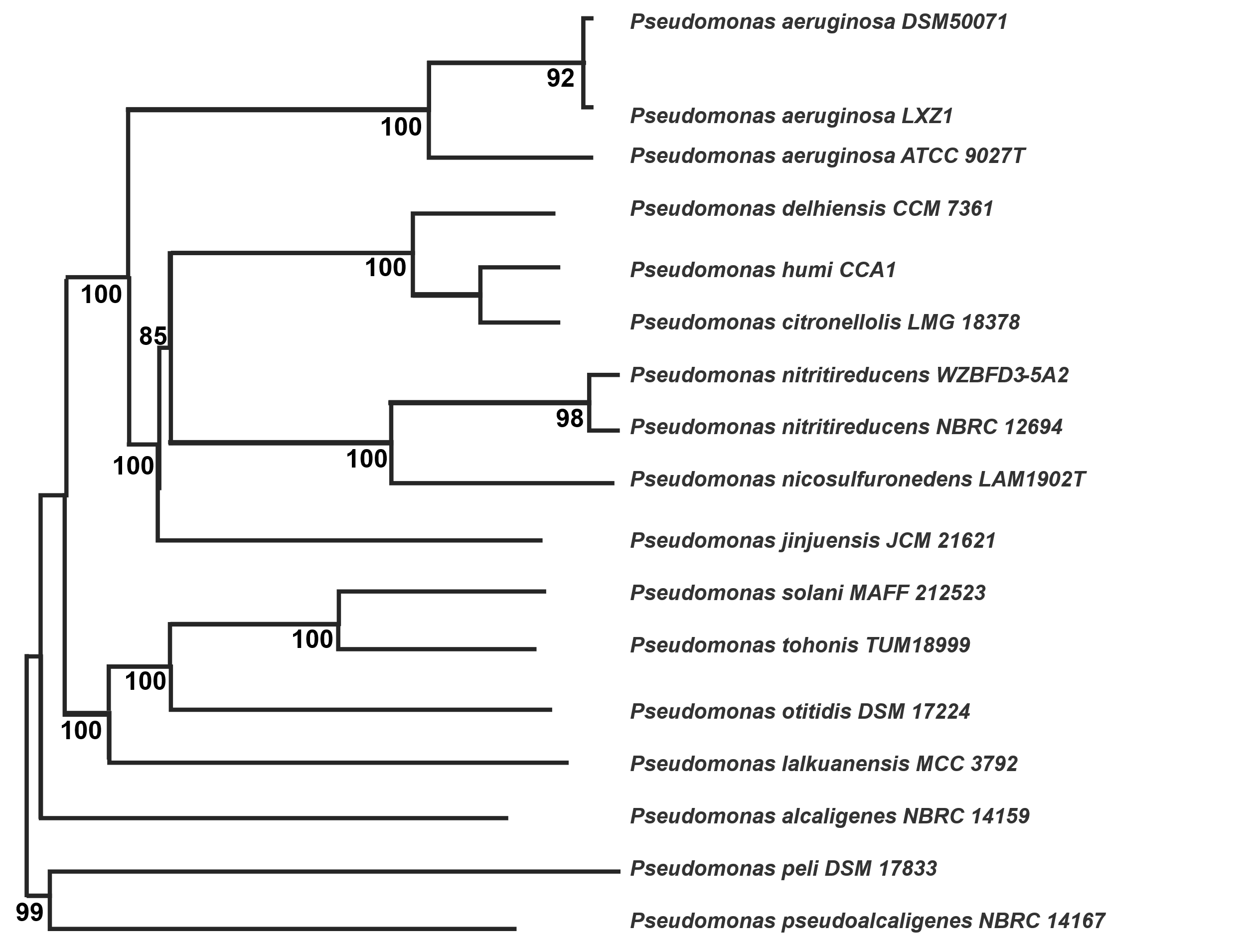


Fig S2. Phylogenetic tree of *Pseudomonas aeruginosa* LXZ1.


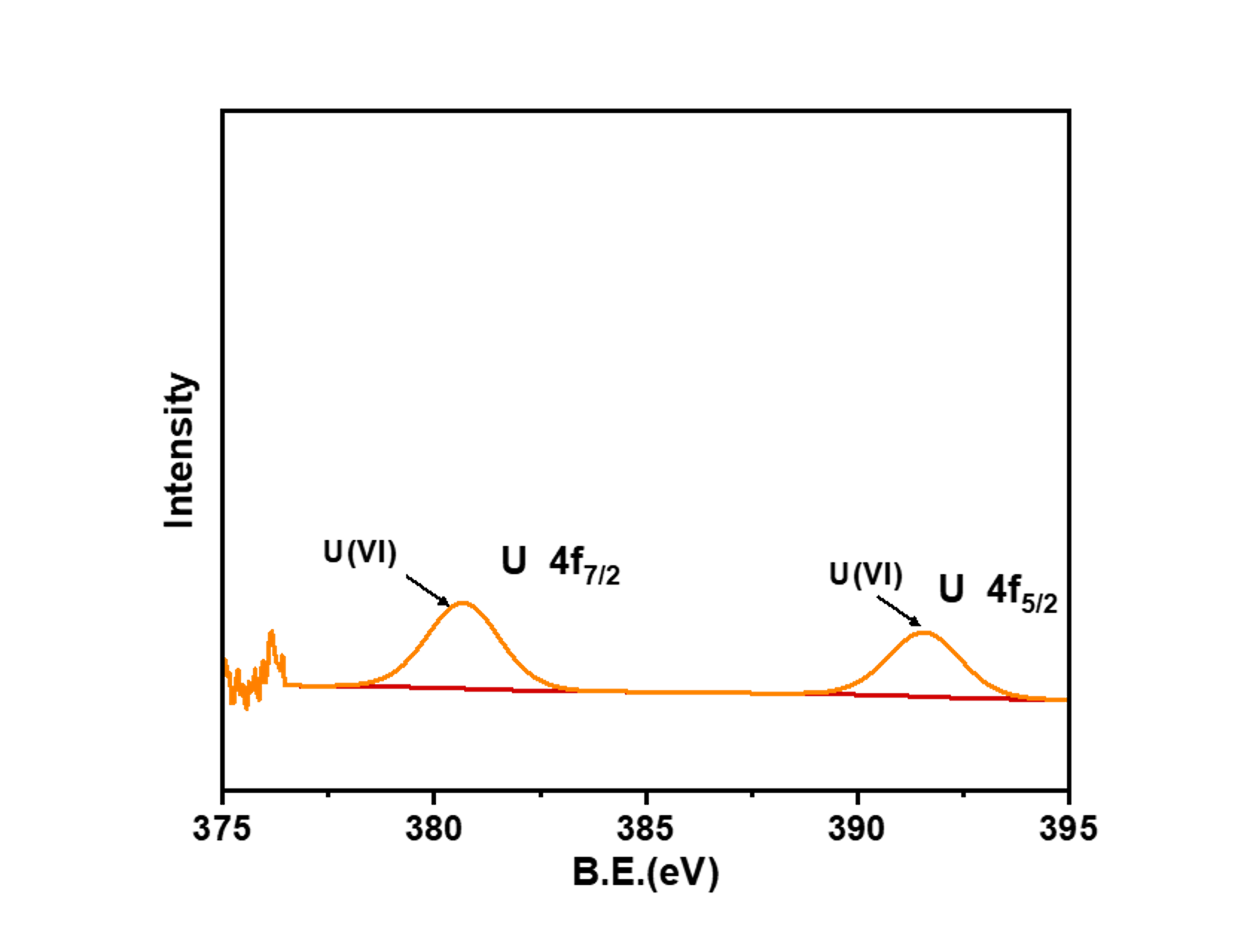


Fig S3. The XPS spectra of P.LXZ1 in the U 4f region were deconvolved using CasaXPS (v2.3.13).


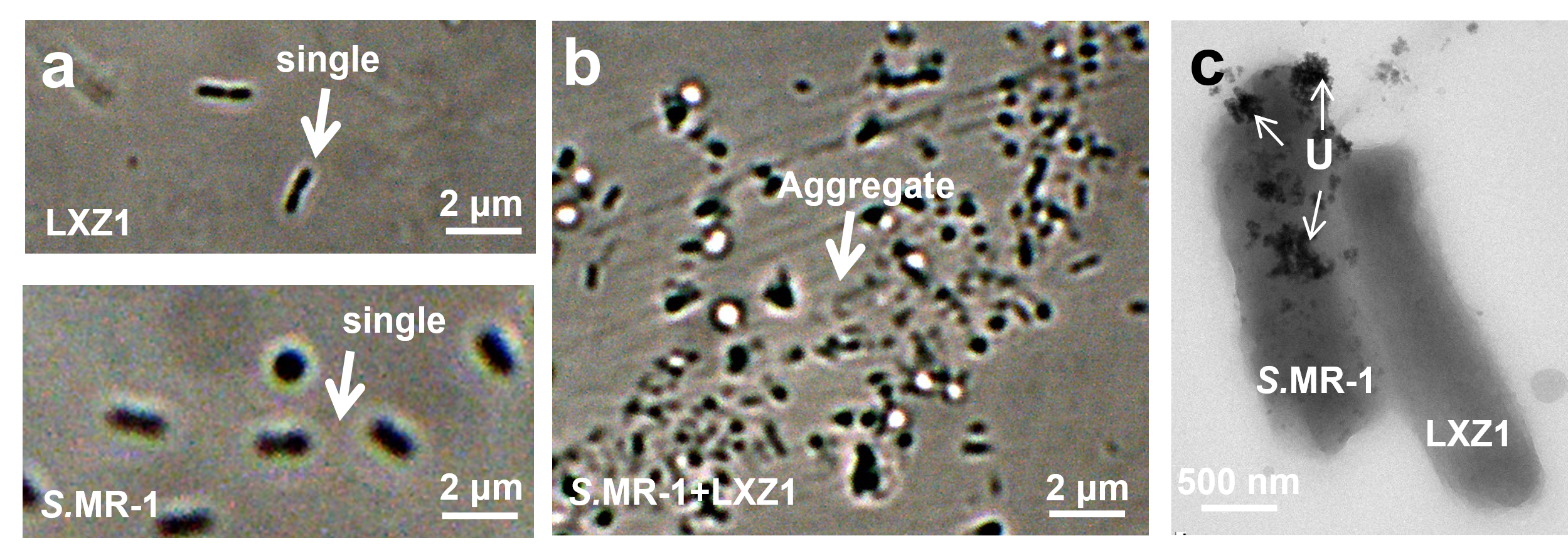


Fig S4. (a-c) Bright field image of S.MR-1, P.LXZ1, and *syncomS+P*. (D) TEM image of *syncomS+P*


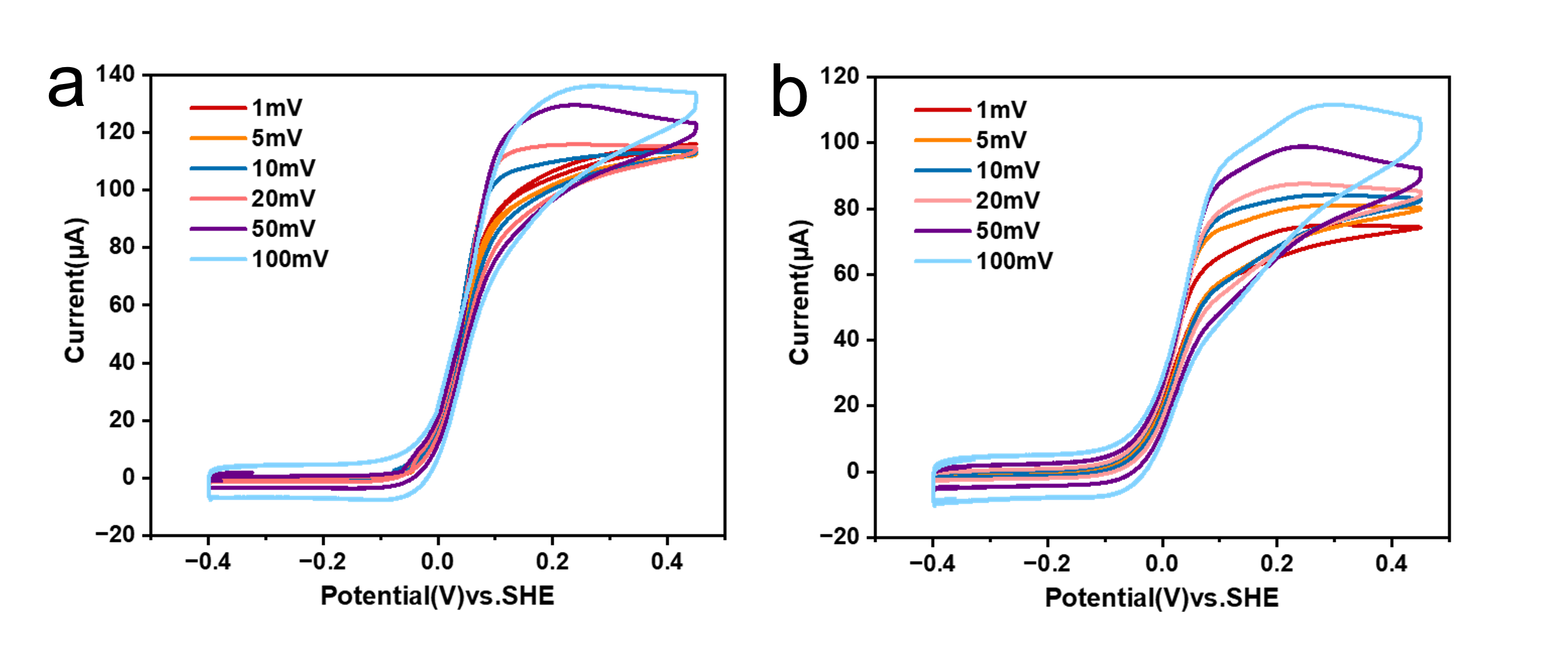


Fig S5. Cyclic voltammetry of (a) *syncomS+P* and (b) *syncomS+P+*DNase I at the scan rate of 1, 5, 10, 20, 50, and 100 mV S^-1^.


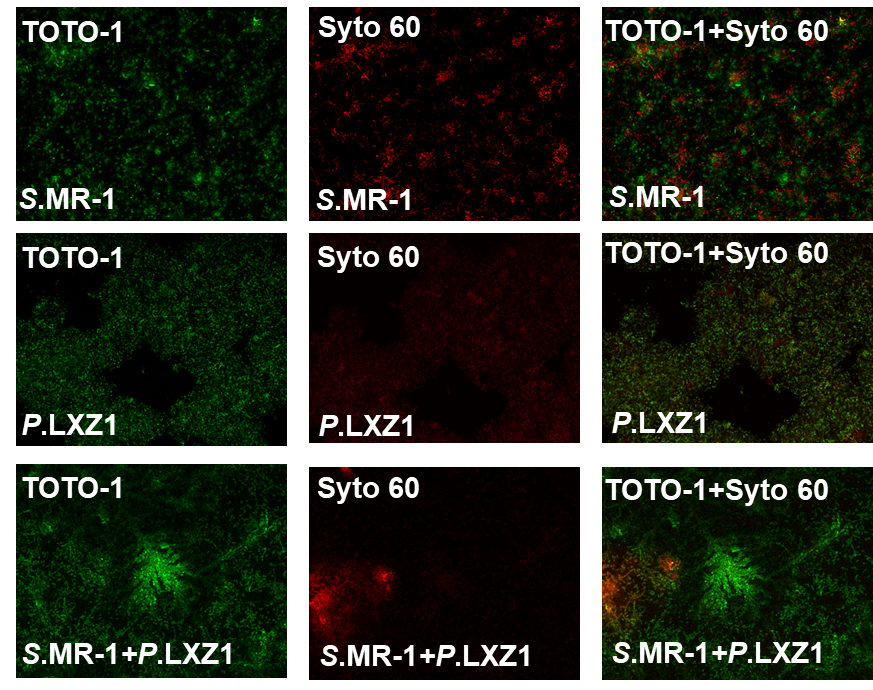


Fig S6. The fluorescent images of *S.*MR-1, *P.*LXZ1, and *syncomS+P* biofilm stained by Syto 60 and TOTO-1.


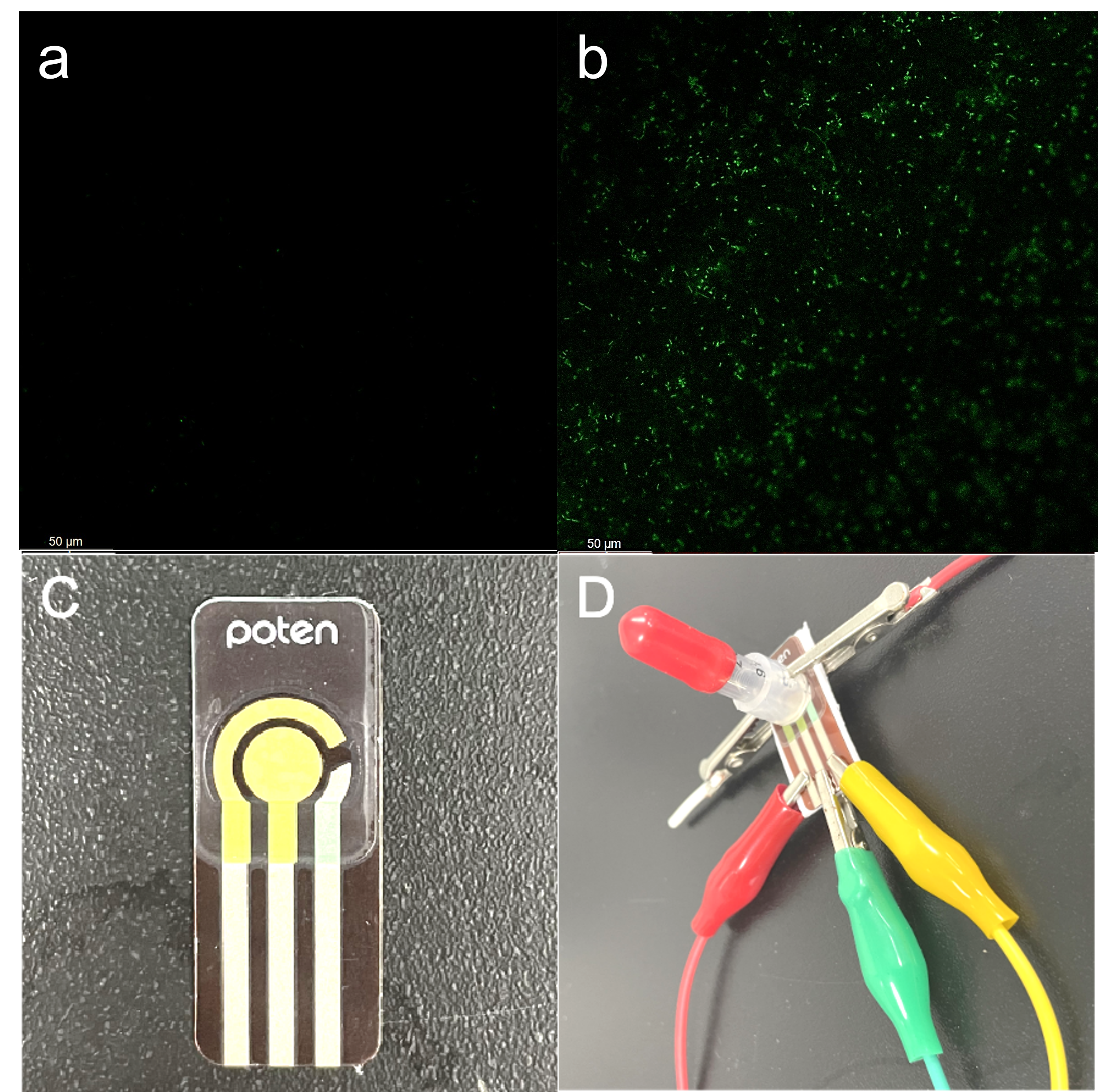


Fig S7. The fluorescence image of (a) gold electrode and (b) that after DNA modification. (c) Gold electrode (d) Schematic diagram of CV measurement for DNA modified electrode.


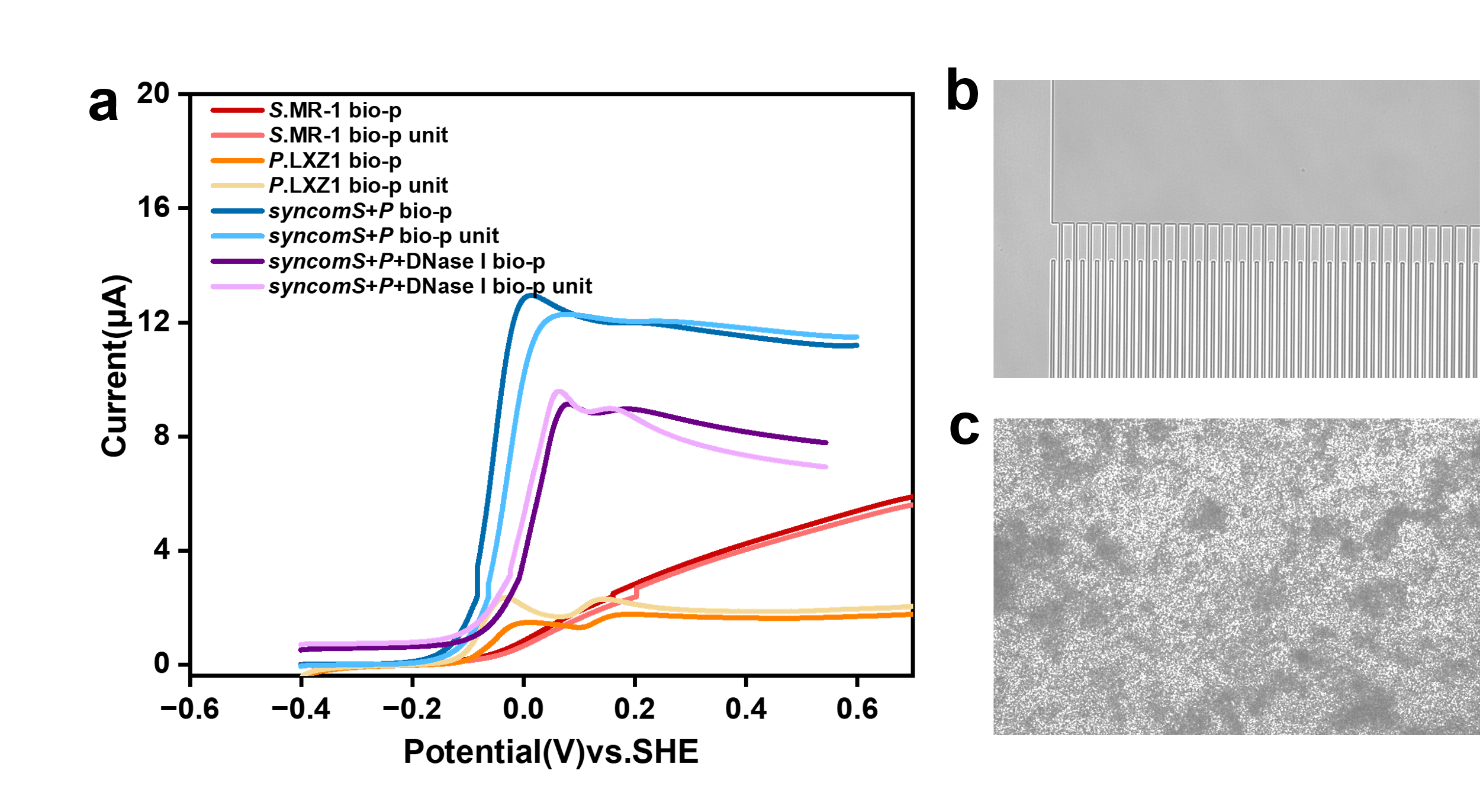


Fig S8. (a) Linear sweeping voltammetry of *S.*MR-1, *P.*LXZ1, and *syncomS+P* biofilm at the scan rate of 1 mV S^-1^ on the interdigitated electrode (IDE). Bright field image of bare (b) IDE and (c) covered with biofilm.


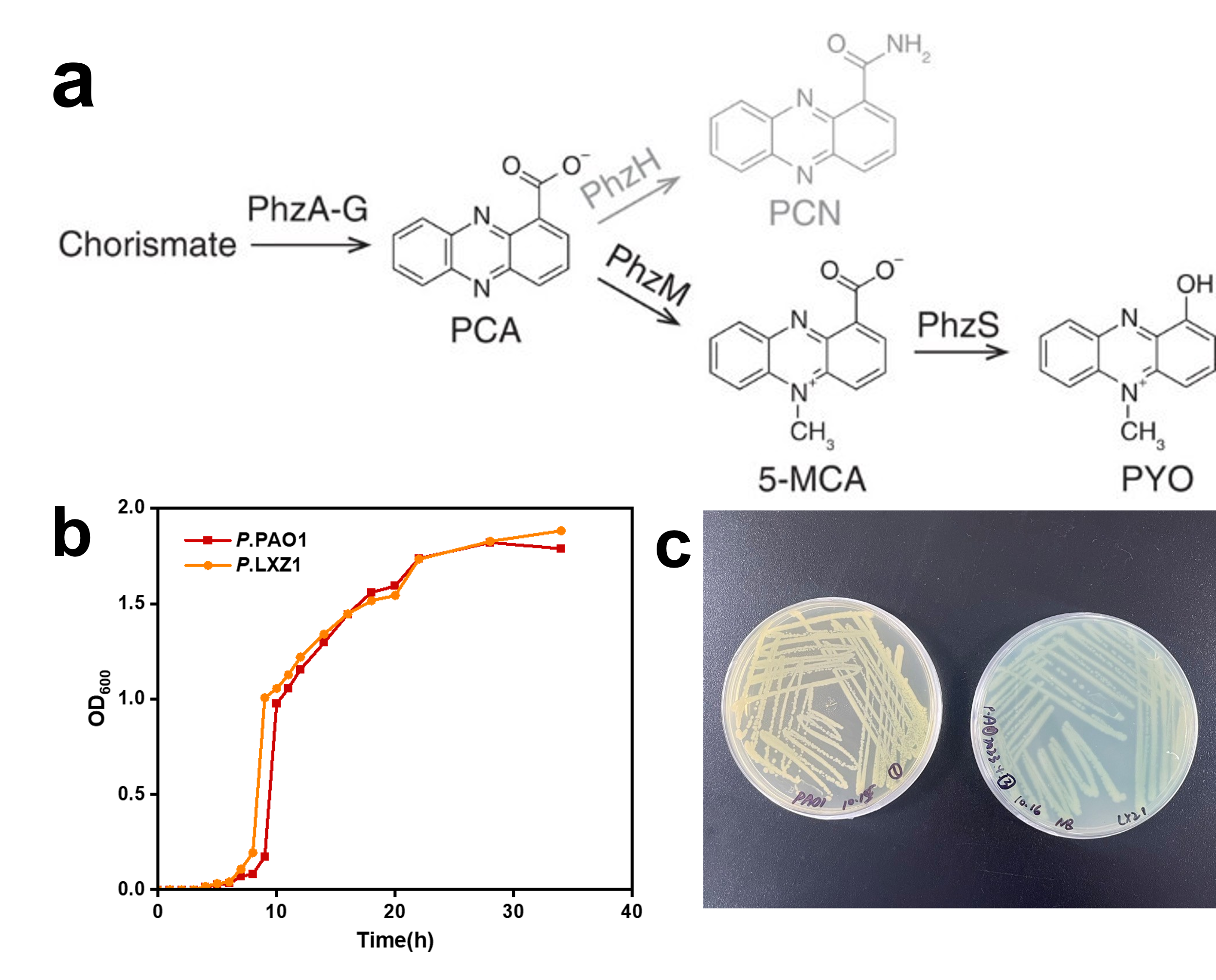


Fig S9. (a) Biosynthetic pathway for phenazine production in *P. aeruginosa*, phenazine-1-carboxylic acid; PCN, phenazine-1-carboxamide; 5-Me-PCA, 5-methyl-phenazine-1-carboxylic acid; PYO, pyocyanin. (b) Growth curves of *Pseudomonas aeruginosa* PAO1 and LXZ1 in LB medium. (c) Streaking plate results of *P. aeruginosa* PAO1 and P.LXZ1.


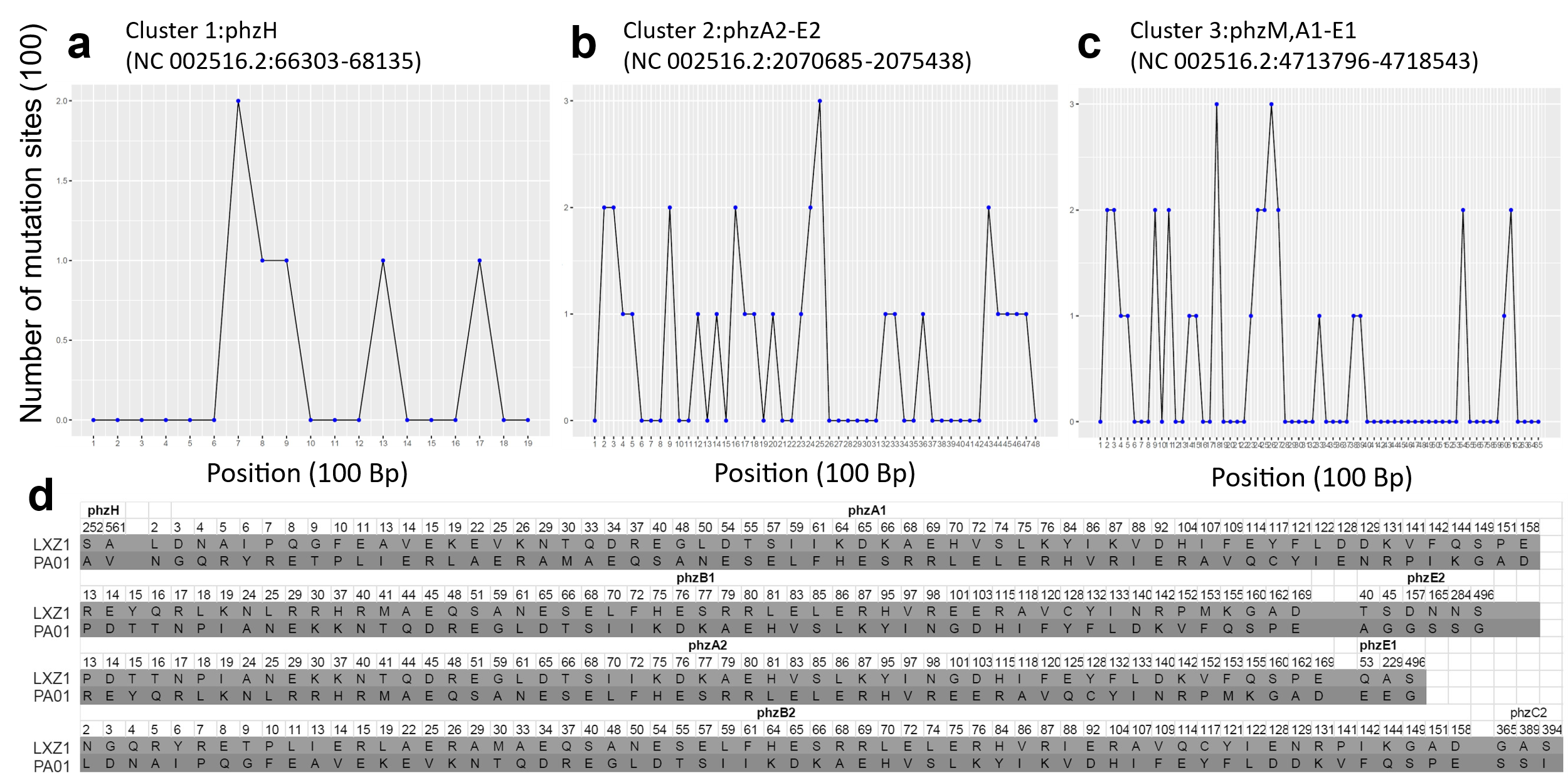


Fig S10. The number of mutation sites of genes between P.PAO1 and P.LXZ1 in (a) Cluster 1:phzH; (b) Cluster 2:phzA2-E2; (c) Cluster 3:phzM,A1-E1. D The mutations of sites of codons in all three clusters.


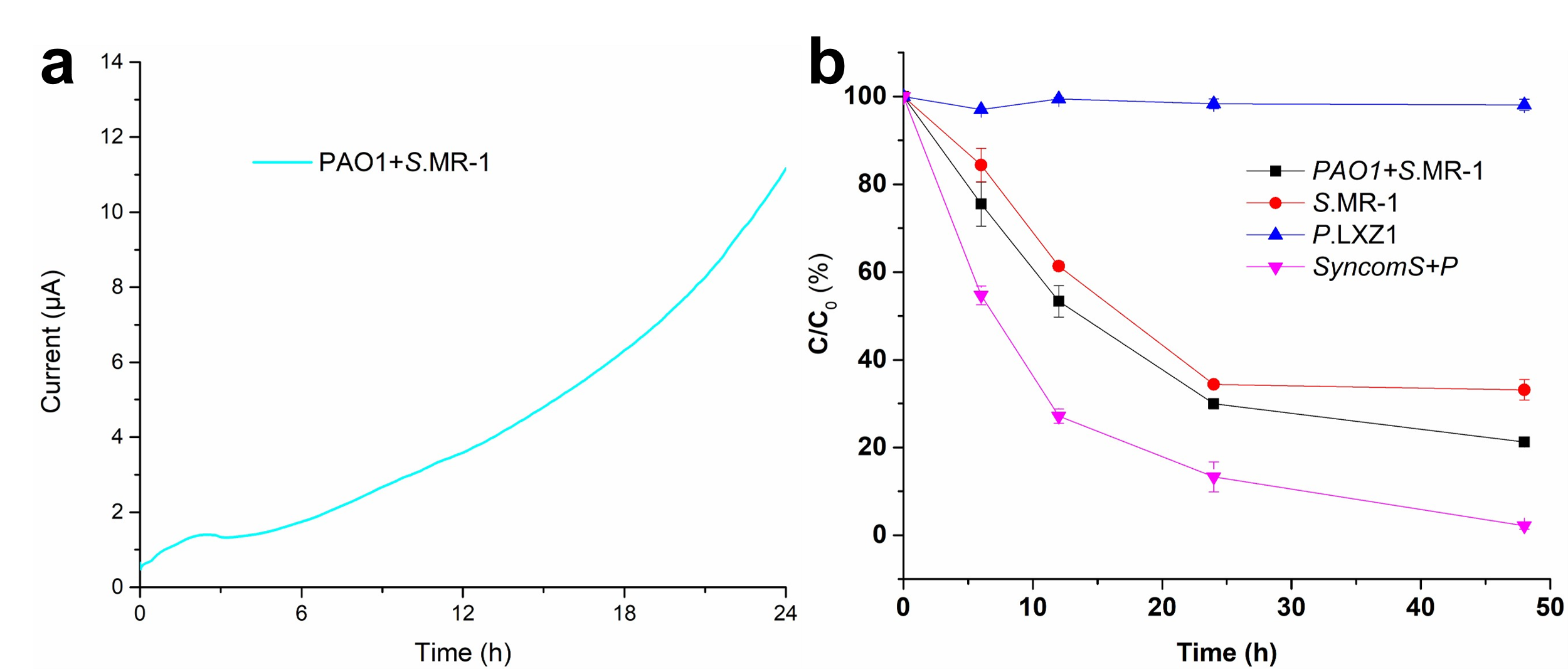


Fig S11. The performance of PAO1 on (A) current generation and (B) U removal.


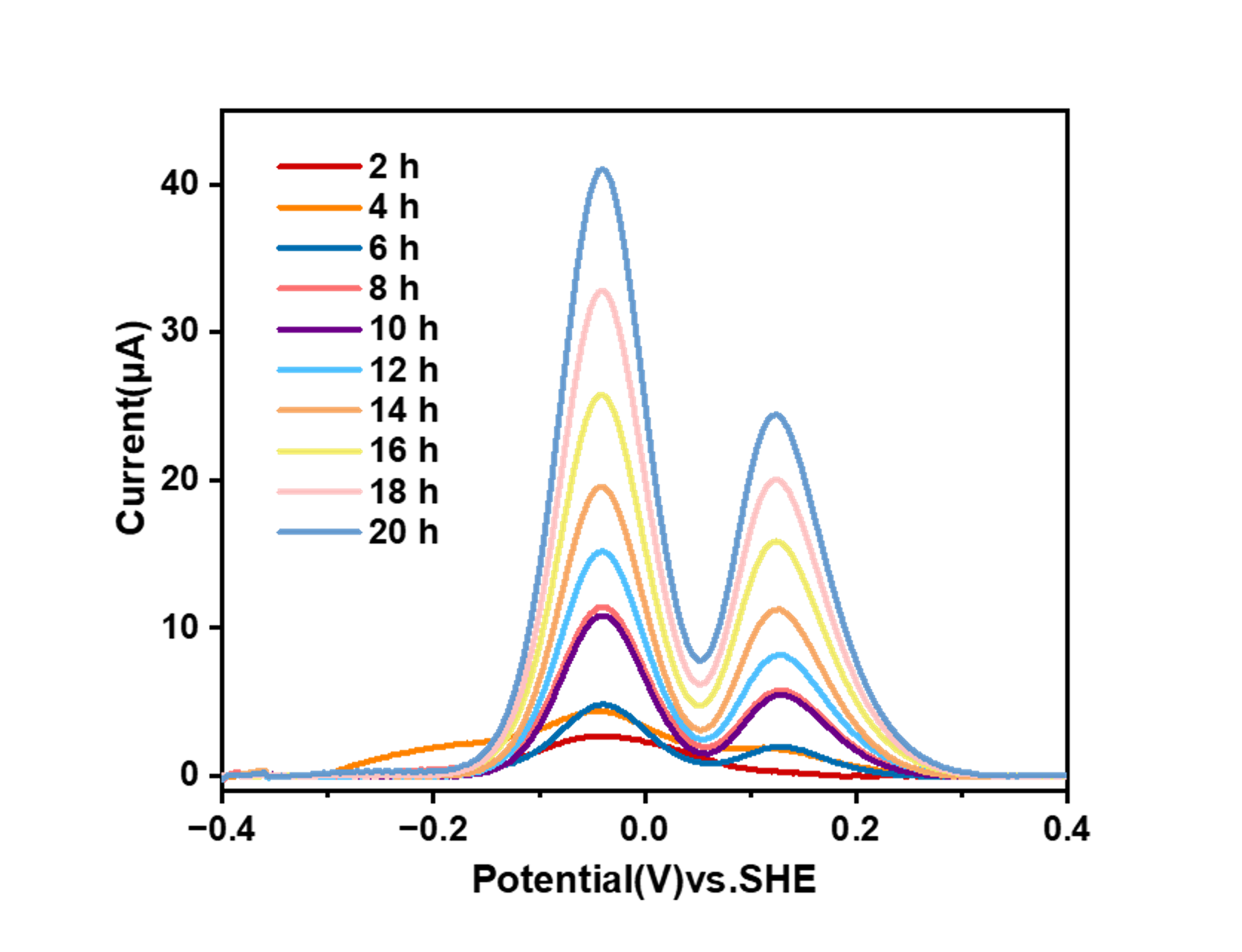


Fig S12. Square-wave voltammograms of *P.*LXZ1 at different times after baseline subtracting.


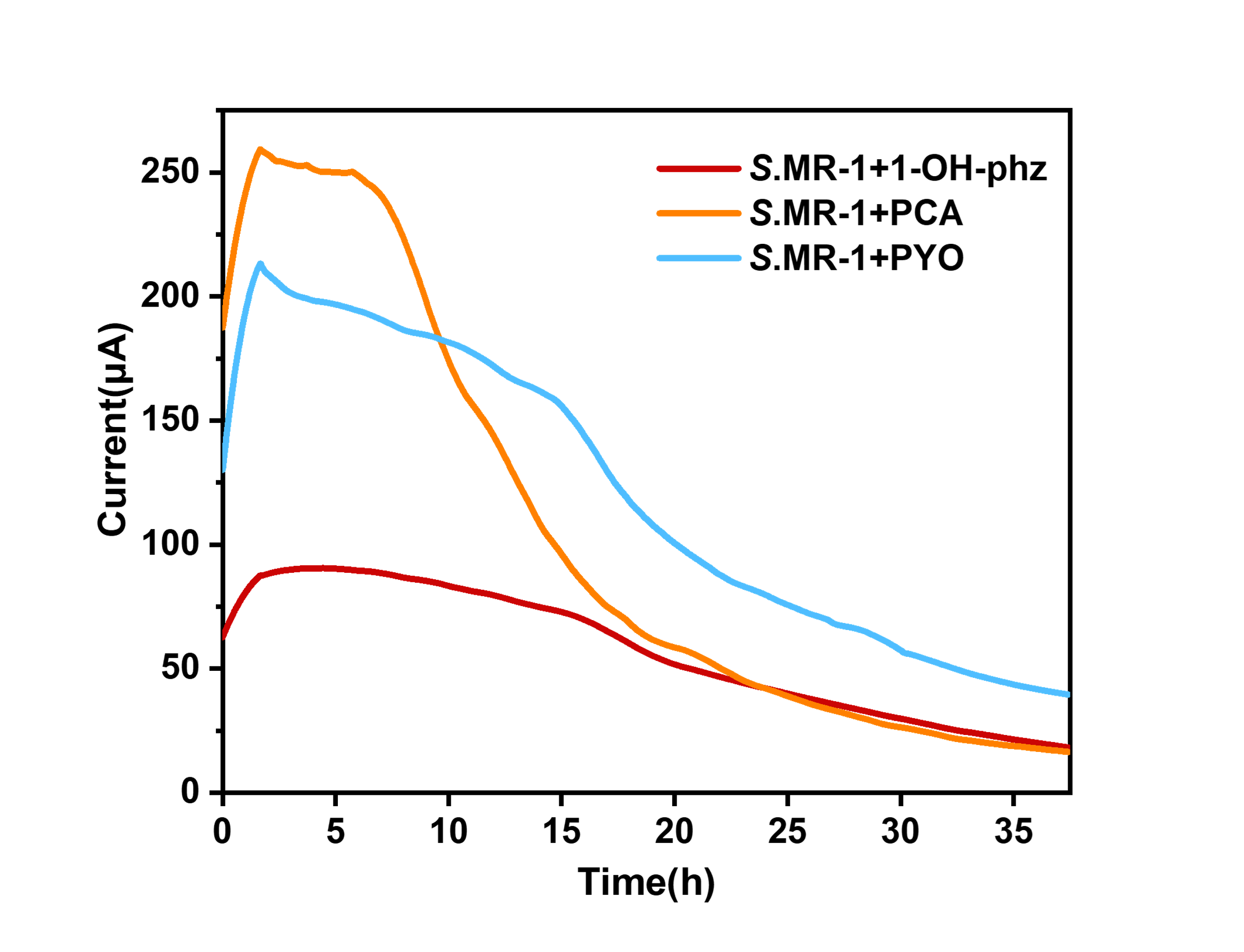


Fig S13. Single-potential amperograms of *S*.MR-1 with 1-OH-phz, PCA, and PYO.


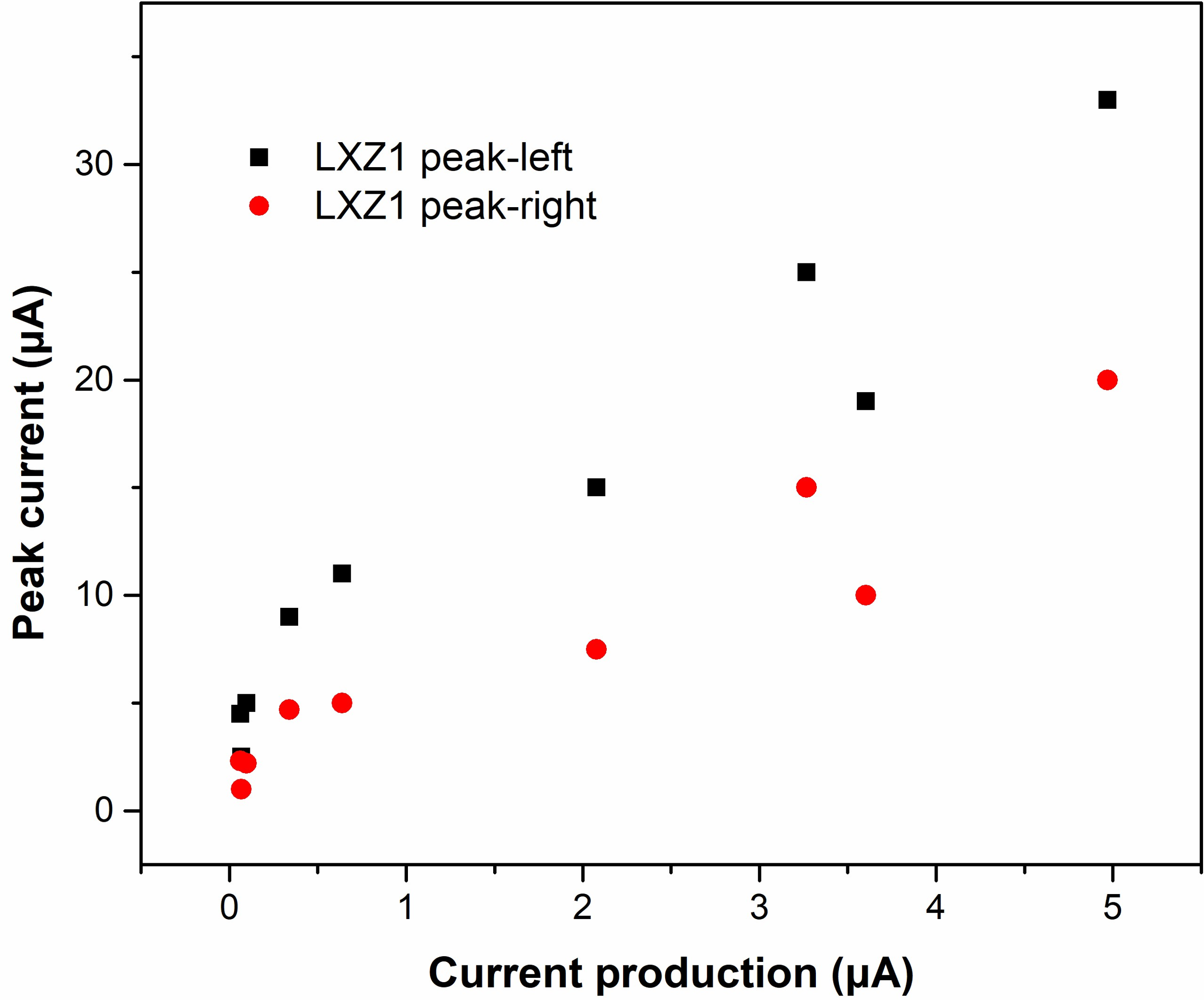


Fig S14. Plot of bacterial current production at an electrode potential of +200 mV (vs. Ag/AgCl KCl saturated) against the peak current of *P*.LXZ1.


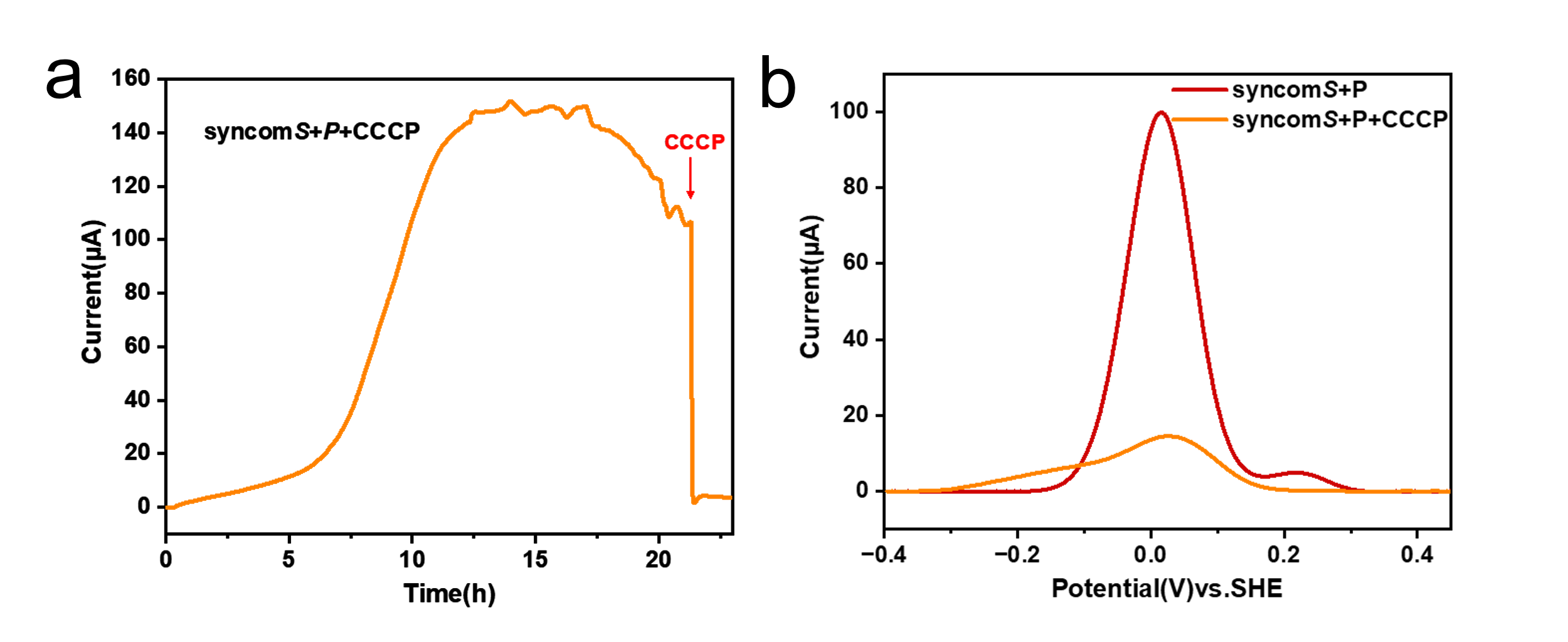


Fig S15. (A) Single-potential amperograms of *SyncomS+P*. (B) square-wave voltammograms of S.MR-1+LXZ1 before and after cccp addtion.


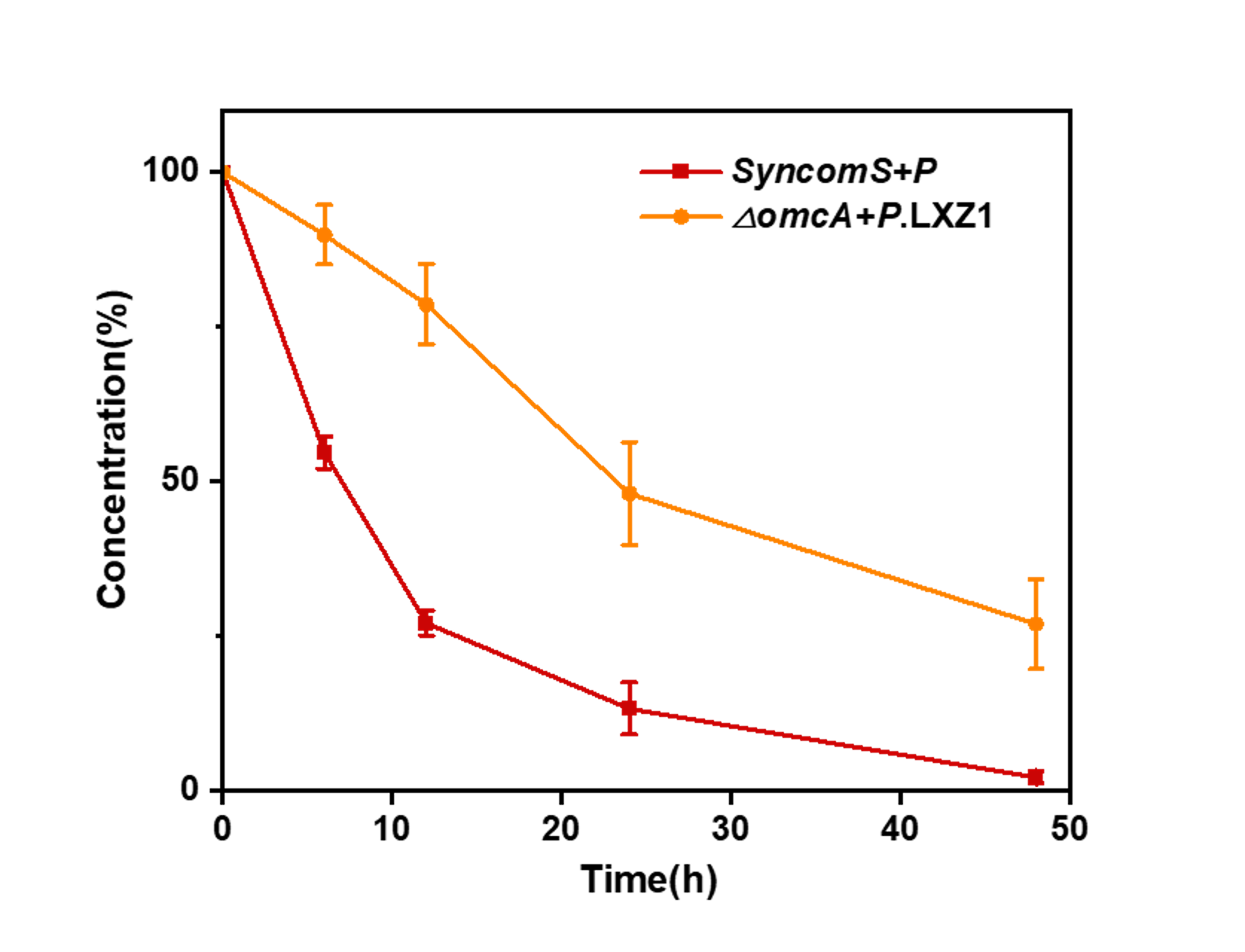


Fig S16. Time course of U(VI) removal by *SyncomS+P* and *ΔomcA*+*P*.LXZ1


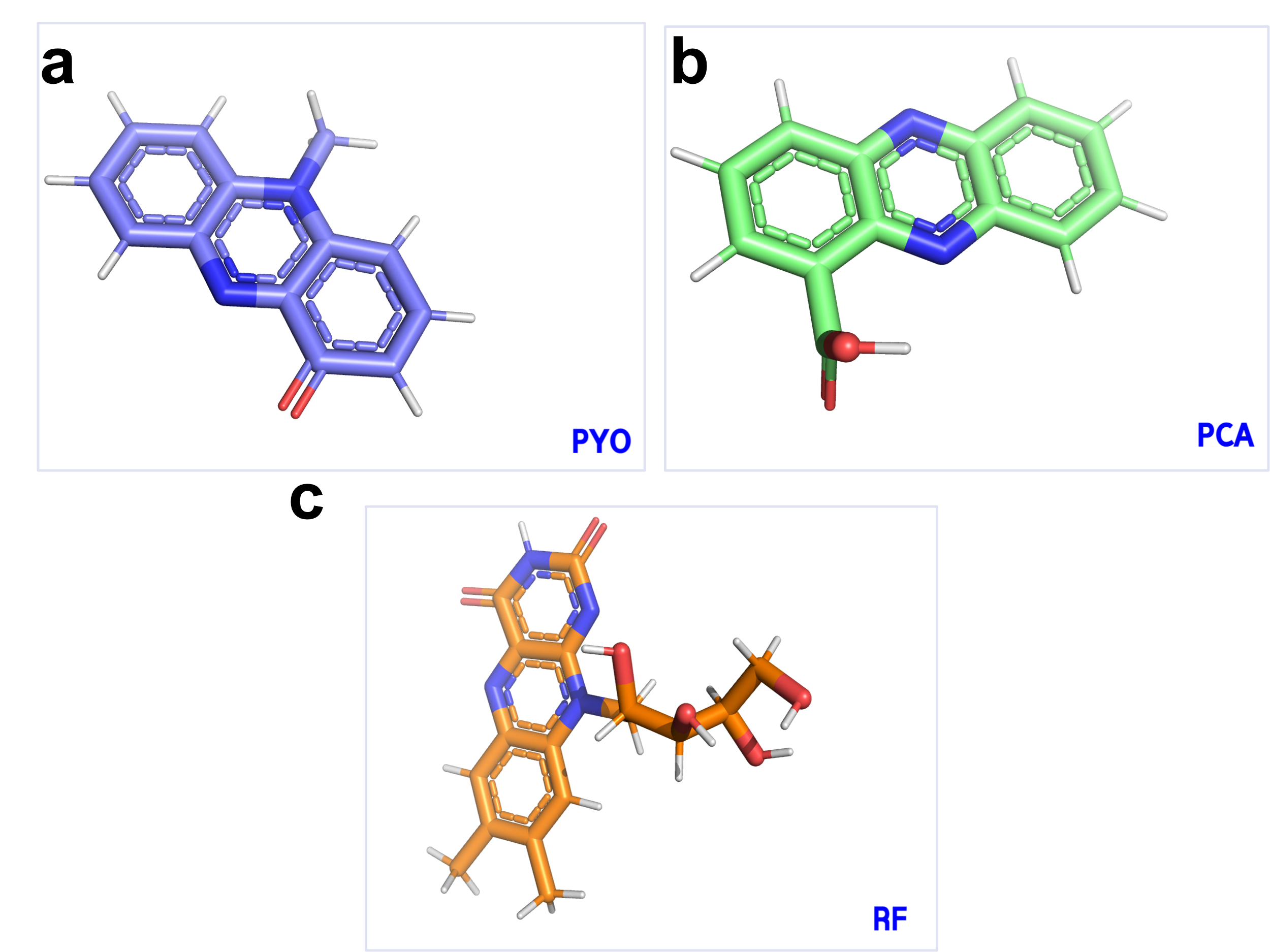


Fig S17. Spatial structure of the molecules (a)PYO, (b)PCA, and (c)RF


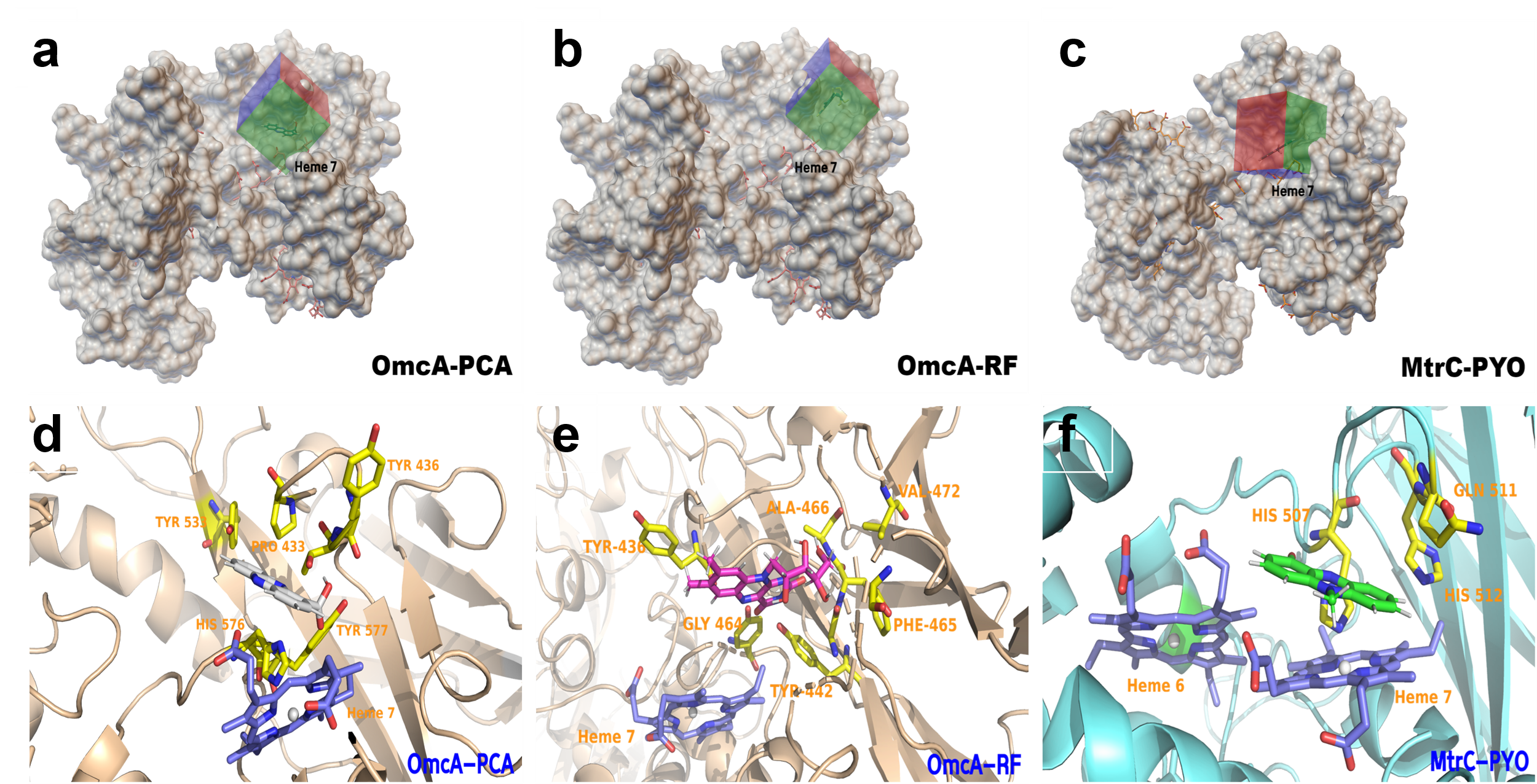


Fig S18. Refined docking simulation of (a)PYO, (b)PCA, and (c)RF with grid map located at the nearest heme, the searching space was centered on heme 7. Molecular dynamics simulations between outer-membrane *c* type cytochromes proteins (*c*-Cyts) (d)OmcA-PCA, (e) OmcA-RF and (f)MtrC-PYO.


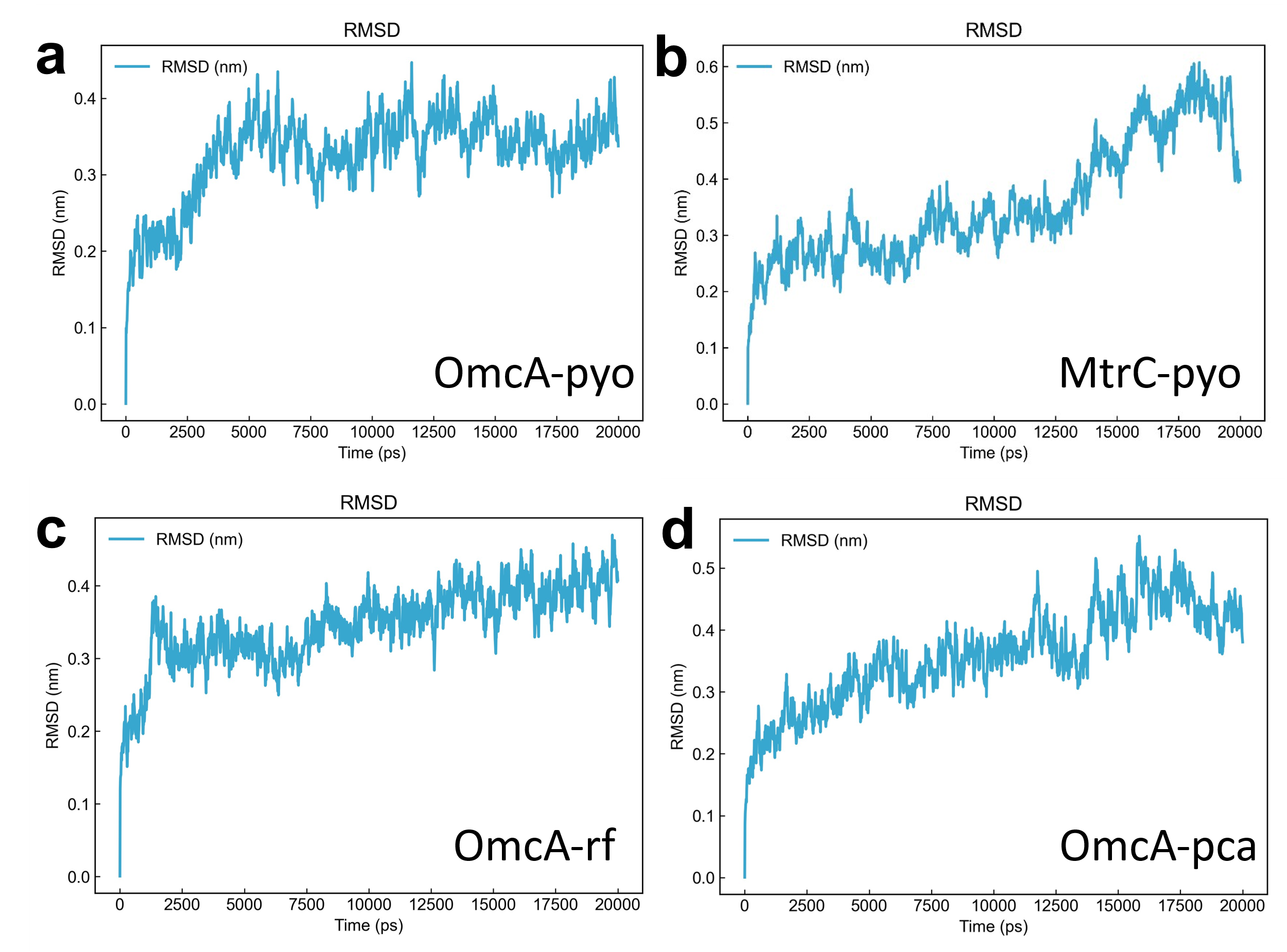


Fig S19. Time course of Root Mean Square Deviation (RMSD) of (a) OmcA-pyo, (b) MtrC-pyo, (c) OmcA-rf, and (d) OmcA-pca.


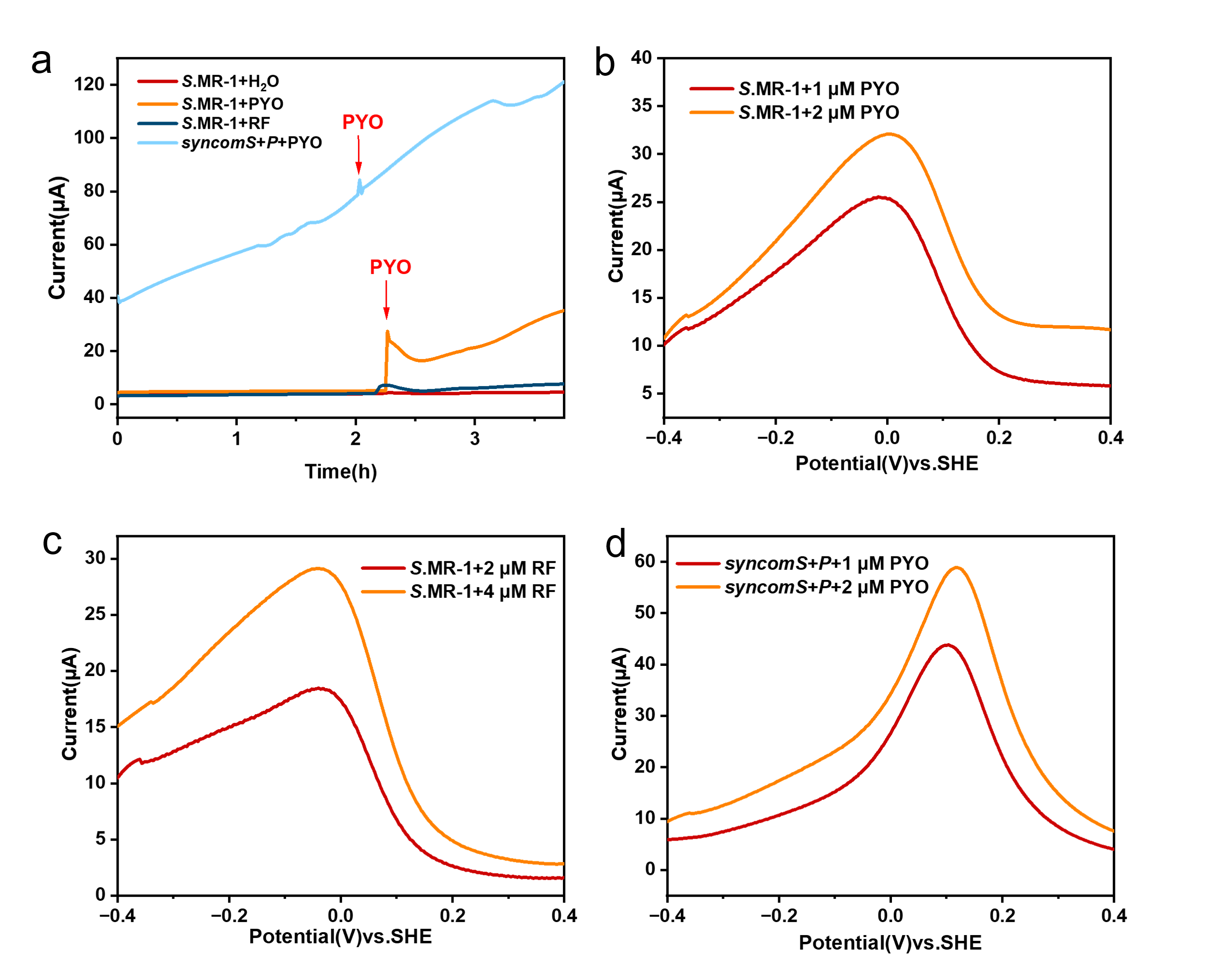


Fig S20. (a)Time profile of current output after adding PYP/RF. Square-wave voltammograms of (b) S.MR-1+PYO, (c)S.MR-1+RF, and (d)*SyncomS+P*+PYO at different times after baseline subtracting.
